# Granulovacuolar degeneration bodies are independently induced by tau and α-synuclein pathology

**DOI:** 10.1186/s13195-022-01128-y

**Published:** 2022-12-14

**Authors:** Marta Jorge-Oliva, Jasper F. M. Smits, Vera I. Wiersma, Jeroen J. M. Hoozemans, Wiep Scheper

**Affiliations:** 1 Department of Functional Genomics, Center for Neurogenomics and Cognitive Research, Faculty of Science, Vrije Universiteit (VU), De Boelelaan 1085, 1081 HV Amsterdam, the Netherlands; 2 Department of Human Genetics, Amsterdam UMC location Vrije Universiteit, De Boelelaan 1117, 1081 HV Amsterdam, the Netherlands; 3 Department of Pathology, Amsterdam UMC location Vrije Universiteit, De Boelelaan 1117, 1081 HV Amsterdam, the Netherlands; 4 Amsterdam Neuroscience, Neurodegeneration, Amsterdam, the Netherlands

**Keywords:** Granulovacuolar degeneration bodies, Tau pathology, α-synuclein pathology

## Abstract

**Background:**

Granulovacuolar degeneration bodies (GVBs) are intracellular vesicular structures that commonly accompany pathological tau accumulations in neurons of patients with tauopathies. Recently, we developed the first model for GVBs in primary neurons, that requires exogenous tau seeds to elicit tau aggregation. This model allowed the identification of GVBs as proteolytically active lysosomes induced by tau pathology. GVBs selectively accumulate cargo in a dense core, that shows differential and inconsistent immunopositivity for (phosphorylated) tau epitopes. Despite the strong evidence connecting GVBs to tau pathology, these structures have been reported in neurons without apparent pathology in brain tissue of tauopathy patients. Additionally, GVBs and putative GVBs have also been reported in the brain of patients with non-tau proteinopathies. Here, we investigated the connection between pathological protein assemblies and GVBs in more detail.

**Methods:**

This study combined newly developed primary neuron models for tau and α-synuclein pathology with observations in human brain tissue from tauopathy and Parkinson’s disease patients. Immunolabeling and imaging techniques were employed for extensive characterisation of pathological proteins and GVBs. Quantitative data were obtained by high-content automated microscopy as well as single-cell analysis of confocal images.

**Results:**

Employing a novel seed-independent neuronal tau/GVB model, we show that in the context of tauopathy, GVBs are inseparably associated with the presence of cytosolic pathological tau and that intracellular tau aggregation precedes GVB formation, strengthening the causal relationship between pathological accumulation of tau and GVBs. We also report that GVBs are inseparably associated with pathological tau at the single-cell level in the hippocampus of tauopathy patients. Paradoxically, we demonstrate the presence of GVBs in the *substantia nigra* of Parkinson’s disease patients and in a primary neuron model for α-synuclein pathology. GVBs in this newly developed α-synuclein/GVB model are induced in the absence of cytosolic pathological tau accumulations. GVBs in the context of tau or α-synuclein pathology showed similar immunoreactivity for different phosphorylated tau epitopes. The phosphorylated tau immunoreactivity signature of GVBs is therefore independent of the presence of cytosolic tau pathology.

**Conclusion:**

Our data identify the emergence of GVBs as a more generalised response to cytosolic protein pathology.

**Supplementary Information:**

The online version contains supplementary material available at 10.1186/s13195-022-01128-y.

## Background

Neurodegenerative proteinopathies share the accumulation of misfolded protein aggregates as key neuropathological feature, which ultimately leads to neuronal loss. Finding therapeutic strategies to halt neurodegeneration in these highly prevalent diseases requires a better understanding of the (early) neuronal response to pathological protein assemblies. Granulovacuolar degeneration bodies (GVBs) are vesicular structures that are found in neurons of tauopathy patients accompanying early pathological accumulations of tau [1–4]. GVBs have strongly been linked to Alzheimer’s disease (AD) given the significantly higher amount of neurons containing GVBs in AD patients compared to age-matched controls [5–7]. Later studies have shown that GVBs are also observed in association with other tauopathies, including frontotemporal dementia (FTD) caused by tau P301L mutation (FTDtau^1^) [3, 8]. Recently, our lab developed the first primary neuron model for GVBs, in which intraneuronal aggregation of human FTDtau^1^ is induced by the addition of K18 tau pre-formed fibrils (PFFs) [9]. GVBs in this live model reproduce key characteristics of GVBs observed in the post-mortem human brain. The model allowed further study of GVBs, demonstrating that they are proteolytically active lysosomal structures that are induced in response to tau pathology. In addition, it was demonstrated that casein kinase 1 isoform δ (CK1δ) is selectively targeted to GVBs. Immunopositivity for a number of other proteins has been found in GVBs, including conflicting results employing several (phosphorylated) tau markers (reviewed in [2]). CK1δ is currently the only cargo validated to accumulate in the GVB core without immunodetection, thus identifying it as a crucial marker for the identification of GVBs. Observations in experimental and post-mortem GVBs led us to propose guidelines for the identification of subcellular structures as GVBs [10], namely the verification of CK1δ in the GVB core in co-localisation with a second established GVB core marker and the presence of a vesicular membrane to demonstrate the characteristic GVB morphology. In this paper, we further refer to structures that could potentially be defined as GVBs, but for which only one or two of these criteria have been confirmed, as “putative GVBs”.

In brains of AD patients, the spatio-temporal spreading pattern of GVBs closely matches that of tau pathology [11]. In addition, there is a strong correlation between local tau pathology and GVB load in GVB-affected brain areas such as the hippocampus [12]. Moreover, studies in the human tauopathy brain have reported the co-occurrence of pathological tau in the same neuron with putative GVBs for which CK1δ positivity has not been demonstrated [1, 4, 13–15] as well as GVBs with a confirmed identity according to our proposed guidelines [16, 17]. This is supported by observations in in vivo and cellular tauopathy models in which GVB identity has been validated [9, 17, 18]. A functional connection between tau pathology and the formation of GVBs is strengthened by experiments in the primary neuron GVB model that show a positive correlation between tau pathology and GVB load [9]. Moreover, experimental GVBs are only present in conditions in which tau aggregates form and not upon FTDtau^1^ overexpression or exposure to tau PFFs alone, demonstrating that cytosolic pathological tau accumulation is required for GVB formation [9].

The strong evidence that connects tau pathology and GVBs is challenged by studies reporting the presence of GVBs [17, 19–23] and putative GVBs for which CK1δ positivity was not demonstrated [3, 13–15, 24, 25] in neurons lacking signal for pathological tau markers (tau−) in the human tauopathy brain. Additionally, the presence of GVBs has also been associated with α-synucleinopathies including Parkinson’s disease (PD) [11, 26–30], PD with dementia [4, 28, 31] and multiple system atrophy (MSA) [4, 30, 32]. The interpretation of these studies is hampered by inconclusive data on GVB identity according to our proposed guidelines, which was only fully confirmed in one of the studies [11], as well as by inconclusive data about the presence of comorbid tau pathology in neurons with α-synuclein (α-syn) pathology (see [10] for a schematic overview of the markers used to assess GVB identity and co-pathology in these studies). However, these observations could indicate that not only tau, but also cytosolic α-syn pathological assemblies elicit GVB formation.

In the present work, we studied the involvement of pathological tau and α-syn in GVB formation. We demonstrate that GVBs are inseparably associated with cytosolic pathological protein assemblies in a newly developed tau/GVB primary neuron model and in post-mortem brain material from tauopathy patients. In addition, we prove the presence of GVBs in a model for α-syn aggregation in cultured primary neurons and in the *substantia nigra* (SN) of PD. In this first α-syn/GVB model, we show that GVB formation is triggered by α-syn pathology in the absence of cytosolic pathological tau accumulation. Interestingly, in addition to positivity for typical GVB markers, GVBs induced by α-syn pathology show an immunoreactivity signature for p-tau epitopes similar to GVBs in the tauopathy context. We conclude that cytosolic pathological protein assemblies of tau and α-syn independently induce GVBs.

## Materials and methods

### Immunolabeling of human brain tissue

Post-mortem brain tissue was obtained from the Netherlands Brain Bank (Amsterdam, the Netherlands; http://brainbank.nl) and the Department of Pathology, Amsterdam University Medical Centers. Donors or their next of kin signed informed consent for brain autopsy, the use of brain tissue and the use of medical records for research purposes. Formalin-fixed, paraffin-embedded brain tissue from the hippocampus or mesencephalon was used for GVB identification and the assessment or quantification of tau or α-syn load in GVB-containing (GVB+) cells via immunofluorescence and immunohistochemistry. For this study, 4 donors (diagnosed with dementia) with Braak stage 4 for neurofibrillary tangles (NFTs) showing abundant tau pathology in the hippocampus and 5 donors (diagnosed with PD) with Braak stage 4 or 5 for Lewy bodies (LB) and showing abundant α-syn pathology in the SN *pars compacta* were selected. Tissue sections with a 5 μm thickness were employed. Double immunohistochemistry was performed to exclude tau and α-syn co-pathology in GVB+ areas (Sup. Fig. 1, Additional file 1).

For immunofluorescence, sections were deparaffinised. Antigen retrieval was subsequently performed by heat pre-treatment (autoclave) in a citrate buffer (0.01 M, pH 6). Sections were incubated overnight (O/N) at room temperature (RT) with primary antibodies (see details in Table 1) diluted in ready-to-use Universal Antibody Diluent Buffer with BSA (Sigma-Aldrich). Negative staining controls consisted in the omission of primary antibodies. For the CK1δ and phosphorylated protein kinase R (PKR)-like endoplasmic reticulum kinase (pPERK) or the lysosomal integral membrane protein 2 (LIMP2) co-staining in the SN, a hippocampal slide from a tauopathy patient was taken along as positive control. Sections were rinsed with phosphate saline buffer (PBS, pH 7.4) before incubation with secondary antibodies. Alexa Fluor (488, 546, 647)-conjugated secondary antibodies (Invitrogen) were diluted 1:400 in ready-to-use Universal Antibody Diluent Buffer with BSA (Sigma-Aldrich) for 2.5 h at RT, after which the sections were rinsed with PBS. To visualise nuclei, sections were stained with 4′,6-diamidino-2-phenylindole (DAPI, Carl Roth) diluted in PBS (6.67 μg/mL) for 10 min at RT followed by PBS washes. To prevent autofluorescence, sections were incubated with Sudan Black (0.2% in 70% ethanol, Sigma-Aldrich) for 10 min at RT and rinsed with PBS. Sections were mounted on coverslips in 80% glycerol in 20% Tris-buffered saline (TBS) and stored at 4°C until imaging.

**Table 1:** Overview of primary antibodies employed

Antibody	Phospho epitope	Species	Dilution			Source	Product number
			Tissue		In vitro		
			IF	IHC			
AT8 (p-tau)	Ser202/Thr205	Mouse monoclonal	1:800	1:200	1:400	Thermo-Scientific	MN1020
AT100 (p-tau)	Thr212/Ser214	Mouse monoclonal			1:200	Thermo-Scientific	MN1060
CK1δ		Mouse monoclonal	1:250	1:250	1:1000	Santa Cruz Biotechnology	sc-55553
CK1δ		Rabbit polyclonal	1:250	1:400	1:500	Thermo-Scientific	PA5-32129
LIMP2		Rabbit polyclonal	1:300		1:500	Novus Biologicals	NB400-129
MAP2		Chicken polyclonal	1:100		1:250	Abcam	Ab5392
MC1		Mouse monoclonal			1:500	Kind gift from Dr. Peter Davies	
pPERK	Thr981	Rabbit polyclonal	1:800		1:1000	Santa Cruz Biotechnology	sc-32577
p-αsyn129 (p-α-syn)	Ser129	Mouse monoclonal	1:100/1:500	1:100	1:2500	Merck	MABN826
p-tau217 (p-tau)	Thr217	Rabbit polyclonal	1:1600		1:100	Thermo-Scientific	44-744

For immunohistochemistry, an extra step after deparaffinisation was performed to quench the endogenous peroxidase activity by incubating the sections with 0.3% H_2_O_2_ in PBS for 30 min at RT. Antigen retrieval and primary antibody (see details in Table 1) incubation were performed as described above. For single stainings, sections were incubated with horseradish peroxidase (HRP)-conjugated goat-anti-mouse secondary antibody (DAKO) for 30 min at RT followed by a washing step with PBS. The sections were then treated with 3,3′diaminobenzidine (DAB) solution (DAB Peroxidase Substrate Kit, Vector Laboratories) according to the manufacturer’s instruction for 10 min at RT and rinsed with water. Nuclei were counterstained with haematoxylin. Sections were dehydrated and mounted using Polyvinyl alcohol mounting medium with DABCO®, antifading (Sigma-Aldrich). For double immunohistochemistry staining, incubation with secondary antibodies HRP-conjugated swine-anti-rabbit (DAKO) and rabbit anti-mouse immunoglobulins/AP (DAKO) was done for 1 h at RT followed by PBS washes. Sections derived from tauopathy patients were then treated with Vector® VIP peroxidase substrate (VECTOR laboratories) for 10 min at RT according to the manufacturer’s instructions. PD patient-derived sections were treated with DAB solution as described above. After washing with PBS, sections were treated with Liquid Permanent Red substrate (DAKO) for 15 min at RT according to the manufacturer’s instructions followed by a washing step in PBS. Sections were subsequently dried for 1 h at 37°C and mounted as described above. Slides were stored in the dark at RT until imaging.

### Animals and primary neuron culture

Animals were housed and bred according to institutional and Dutch governmental guidelines. Experiments were approved by the animal ethical committee of the VU University/VU University Medical Center.

Cerebral cortices from embryonic day 18.5 wild type (WT) C57BL/6 mice were dissected and disposed of meninges in ice-cold Hanks’ balanced salt solution (Sigma-Aldrich) containing 10 mM HEPES (Gibco; Hanks-HEPES). The isolated tissue was digested by 0.25% trypsin (Gibco) in Hanks-HEPES for 20 min at 37°C. Digested tissue was washed three times in Hanks-HEPES and triturated with fire-polished Pasteur pipettes in DMEM (Lonza) supplemented with 10% heat-inactivated foetal bovine serum (Gibco), 1% penicillin-streptomycin (Gibco) and 1% non-essential amino acid solution (Gibco). Dissociated cells were spun down, resuspended and plated in neurobasal medium (Gibco) supplemented with 2% B-27 (Gibco), 18 mM HEPES, 0.25% Glutamax (Gibco) and 0.1% penicillin-streptomycin (NB+).

Plates and glass coverslips were coated with 5 μg/mL poly-l-ornithine (Sigma-Aldrich) and 2.5 μg/mL laminin (Sigma-Aldrich) O/N at RT. Neurons were plated at a density of 15,000 or 40,000 cells/well in 96- or 24-well plates, respectively.

Cells were maintained at 37°C, 5% CO2. Fresh NB+ medium (40% of the total volume in the well) was supplied to the cultures after 10 days in vitro (DIV 10).

### Tau transduction in primary neurons

Lentiviral transductions were performed on WT neurons using 2N4R human P301L, S320F tau (FTDtau^1+2^) or FTDtau^1^. FTDtau^1^ or untransduced cells were employed as control. All constructs were cloned in second-generation lentiviral backbone vectors under the cytomegalovirus promoter. Lentiviral particles were generated as described previously [33], aliquoted and stored at −80°C until use.

### Induction of α-synuclein aggregation in primary neurons

Recombinant full-length human WT α-syn with a N-terminal His-tag and a C-terminal Avi-tag was obtained as previously described [34]. To prepare PFFs, 0.9 mg/mL recombinant protein in PBS was incubated at 37°C with constant agitation for 4 days. The presence of cross-β-sheet structure and final plateau of aggregation was verified by Thioflavin T fluorescence. The reaction product was centrifuged (20000 g) at 4°C for 1 h and the supernatant was discarded. The pellet was resuspended in PBS (1.67 mg/mL) and sonicated with a Hielscher UP200St ultrasonic homogeniser (two cycles of 1 min in pulses of 10 s and 5 s rest). The resulting PFFs were aliquoted, snap frozen and stored at −80°C. Commercial untagged human WT α-syn PFFs (StressMarq) were also employed. Prior to use, PFFs were further diluted in PBS into the required concentration and re-sonicated (1 min in pulses of 10 s and 5 s rest). PFFs were directly added to the medium of WT neurons to reach a final concentration of 5 μg/mL. Equal volume of PBS was added as control condition.

### Fixation of primary neurons

Cells were fixed at DIV 18 in a two-step fixation protocol. An equal volume to culture media of formaldehyde (Electron Microscopy Sciences; FA) in PBS (pH 7.4) was added to neurons to reach a final concentration of 1.85% for 10 min at RT followed by 10-min incubation with 3.7% FA at RT.

In some experiments, indicated in the text, cells were fixed in ice-cold methanol (MeOH) for 15 min at −20°C while gently shaking to remove soluble proteins.

After fixation, cells were washed and stored in PBS (pH 7.4) at 4°C.

### Immunolabeling of primary neurons

Fixed cells were permeabilised in 0.5% Triton X-100 (ThermoFisher Scientific) in PBS (pH 7.4) for 5 min at RT and blocked in 2% normal goat serum (Gibco) and 0.1% Triton X-100 in PBS (blocking solution) for 30 min at RT. Primary antibodies (see details in Table 1) were diluted in blocking solution and incubated O/N at 4°C. Alexa Fluor (405, 488, 546, 647)-conjugated secondary antibodies (Invitrogen) were 1:500 diluted in blocking solution. Cells were washed with PBS and incubated with secondary antibody solution for 1 h at RT or for 2 h in the case of 405-conjugated secondary antibodies. Cells were rinsed with PBS. To visualise nuclei, cells were incubated with a solution of DAPI (Brunschwig Chemie) diluted in PBS (5 μg/mL) at RT for 5 min followed by a PBS wash. At this point, 96-well plates were stored in the dark at 4°C until imaging. Coverslips were mounted on microscope slides (ThermoFisher Scientific) using Mowiol (Sigma-Aldrich). The mounted slides were kept in the dark and left to dry O/N at RT. Slides were stored in the dark at 4°C until imaging.

### Imaging of immunolabeled tissue and primary neurons

Immunofluorescent imaging was performed using a Nikon Eclipse Ti confocal microscope controlled by NisElements 4.30 software (Nikon). A 60× oil immersion objective (NA = 1.4) or a 40× oil immersion objective (NA = 1.3) was employed. Z-stacks with a step size of 0.5 or 1 μm were acquired unless indicated otherwise.

Immunohistochemical stainings were imaged using a Leica DM2500LF microscope equipped with a 10× air objective (NA = 0.32) controlled by Las V4.12 software (Leica) and a Leica DM5000B microscope equipped with a 60× oil objective (NA = 1.4) controlled by Las V4.4 software (Leica). Single focal plane images were acquired.

In all cases, laser settings were adjusted to prevent saturation in any of the channels. Laser settings were kept constant within independent experiments to allow valid comparison of different conditions. For imaging of GVBs and tau pathology markers in SN or in the α-syn model, a hippocampal slide from a tauopathy patient or neurons from the FTDtau^1+2^ model, respectively, were taken along to set imaging parameters.

### Confocal microscopy image analysis

Confocal images were analysed using ImageJ software (National Institutes of Health). Neurons with ≥2 CK1δ or pPERK puncta were classified as GVB+ neurons, while neurons with no puncta were considered GVB−. Unless stated otherwise, maximum intensity projected Z-stacks are shown. The number of independent experiments or patients and number of cells analysed are indicated in the figure legend and/or in Sup. Table 1, Additional file 1.

For single-cell tau quantification of GVB+ neurons in human brain tissue and primary cultures, GVB+ neurons were selected without knowledge of their tau pathology status. For comparison of tau pathology load between GVB− and GVB+ neurons in the tau/GVB model, neurons were selected without knowledge of their GVB status. Only neurons with a clear microtubule-associated protein 2 (MAP2) signal were included in the analysis. In the case of patient tissue, the full Z-stack was screened for tau signal and single focal planes were used for analysis to prevent quantification of signal in other planes pertaining to surrounding tissue. Selected GVB− neurons that visually contained no tau signal were employed as control neurons in the analysis (tau−/GVB−). In the case of GVB+ neurons, single focal planes in which GVBs and tau signal, if detected, were visible were selected for further analysis (tau?/GVB+). For primary neurons in culture, isolated neurons were selected and maximum intensity projected Z-stacks were employed. Tau−/GVB− neurons were selected from the control conditions, and tau?/GVB+ neurons were selected from the experimental conditions solely based on the GVB status. Per neuron, a somatic mask was drawn manually based on the neuron-specific MAP2 signal and was selected as the region of interest (ROI) for quantification. Thick, proximal neurites were also included in the mask for quantification in the tauopathy brain. Mean tau intensity as well as maximum tau intensity were determined for each neuron. Corrected mean tau intensity was obtained by calculating the ratio with the average mean tau intensity value in GVB−/tau− per patient or experiment. Maximum intensity values were corrected by subtracting the average mean tau intensity of GVB−/tau− per patient. The corrected mean intensity was Log2 transformed.

Line segments were drawn across CK1δ puncta to visualise intensity profiles for CK1δ and additional GVB markers. CK1δ and pPERK co-stainings were analysed in maximum intensity projections while CK1δ and LIMP2 co-stainings were analysed in single focal planes to better visualise the membrane signal. Fluorescence intensity is shown in arbitrary units.

### High-content microscopy and automated analysis

Primary neurons cultured in 96-well plates were imaged on a CellInsight CX7 High-Content Screening (HCS) Platform (ThermoFisher) controlled by HCS Studio Cell Analysis software (ThermoFisher). Widefield mode and a 20× air objective were employed. Z-stacks with a step size of 3 μm were acquired for CK1δ in order to include all GVBs and a single focal plane was acquired for the rest of the channels. Forty randomly distributed fields were imaged per well. DAPI signal was used for autofocus in all cases.

Maximum intensity projections were analysed with Columbus analysis software (v2.5.2.124862; PerkinElmer) by in-house developed scripts. Single well values representing the average of 40 fields were used for analysis. Neuronal nuclei were identified by the DAPI signal and distinguished from non-neuronal nuclei by size and overlap with the neuron-specific marker MAP2. The number of neurons was extracted from this quantification. Tau pathology load was determined by MC1 signal measured in a MAP2-defined ROI. GVB load was determined using an adaptation of the protocol employed in [9]. In brief, CK1δ images were filtered to remove smooth and continuous background intensity. CK1δ puncta were selected based on intensity and clustered when they were in close proximity. Selection of clusters to determine the number of neurons with GVBs was made based on the area, roundness and intensity of the cluster in addition to their overlap with MAP2. All parameters were corrected for the number of neurons per well and subtracted the average (background) signal measured in control wells per experiment. Mean corrected values corresponding to the longest treatment duration with FTDtau^1+2^ were set to 100% per experiment and the rest of values were re-scaled accordingly. In the graph, mean corrected values per condition are shown. Two replicate wells were included per condition per experiment and data from 3 independent experiments were pooled together. Data points represent single well observations.

### Statistics

RStudio version 4.0.2 (RStudio) software was used for creating density plots. Binwidth was adjusted per dataset to achieve a smooth curve: in the case of Fig. 3c, it was set at 0.05 for tau−/GVB− neurons and at 0.5 for tau?/GVB+ neurons. For Fig. 2e, binwidth was set at 0.2 for tau−/GVB− neurons and at 0.5 for tau?/GVB+ neurons. Binwidth for all datasets in Fig. 6c was set at 0.5. Graphpad Prism version 8.4.2 (Graphpad) software was used for statistical analysis and visual representation of the rest of the data. The Shapiro-Wilk test was used to assess the normality of data distribution. Nested one-way ANOVA with Tukey’s post hoc test for multiple comparison and Kolmogorov-Smirnov test were used to test densities of normally and not normally distributed data, respectively. Nested *t*-test and non-parametric Mann-Whitney *U* test were used for statistical analysis of two groups of normally and not normally distributed data, respectively. Nested one-way ANOVA with Dunnett’s post hoc test was used for analysis of more than two groups in comparison to a control dataset. A *p* value < 0.05 was considered statistically significant. Statistical tests performed are indicated in the figure legends and *p* values are shown in the graphs as *p < 0.05, **p < 0.01, ***p < 0.001, ****p < 0.0001 and n.s*.* not significant.

## Results

### Seed-independent intracellular tau aggregation induces GVB formation

**Fig. 1:**
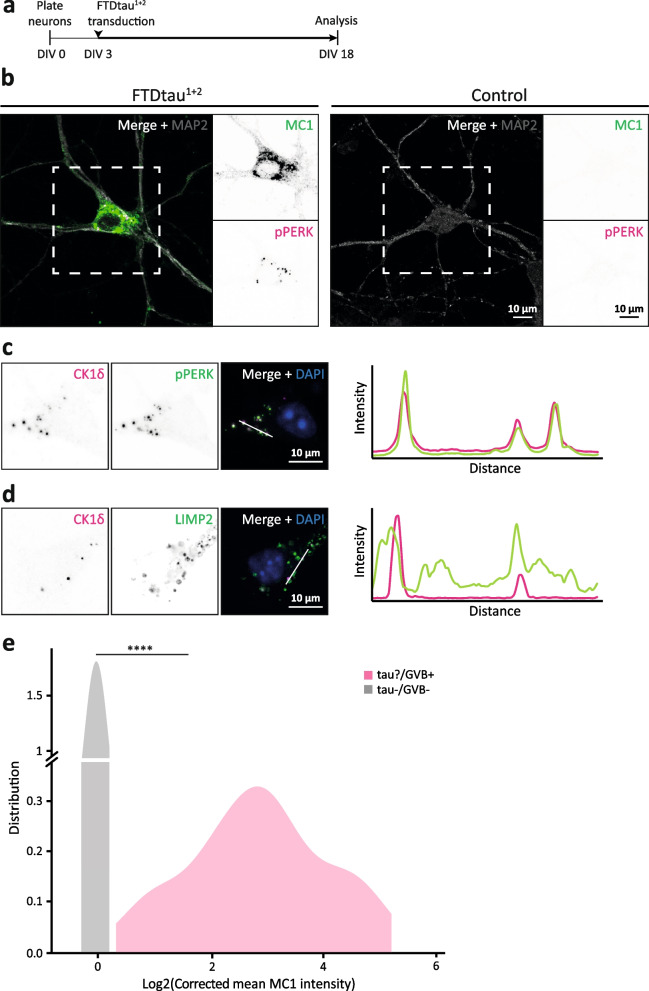
Pathological tau accumulation is a prerequisite for GVB formation in the FTDtau^1+2^ model. **a** Schematic representation of the protocol to induce seed-independent intraneuronal tau pathology in primary mouse neurons. WT neurons were plated at DIV 0. At DIV 3, neurons were transduced with FTDtau^1+2^. Cells were fixed and analysed by immunofluorescent staining for pathological tau and GVBs at DIV 18. **b** Representative confocal images of FTDtau^1+2^-transduced tau+/GVB+ neuron (left) and a tau−/GVB− untransduced neuron (right) shown as control. Immunofluorescence staining was performed for the neuron-specific dendritic marker MAP2 (grey), MC1 (green) and pPERK (magenta). Separate channels are shown in greyscale. **c**, **d** Representative confocal images of GVB+ neurons in the FTDtau^1+2^ model. Co-immunofluorescence staining was performed for the canonical GVB marker CK1δ (magenta) and the additional GVB core marker pPERK (**c**) or the GVB membrane marker LIMP2 (**d**) (green). Nuclei are visualised by DAPI (blue). Separate channels are shown in greyscale and in colour in the merge. A line intensity profile along the white bar indicated in the merge shows CK1δ co-localisation with pPERK and that is surrounded by LIMP2 as additional GVB markers. **e** Density plot of single-cell somatic Log2-transformed corrected mean MC1 immunofluorescence intensity of tau−/GVB− (grey) and tau?/GVB+ (pink) neurons in the FTDtau^1+2^ model. *N*=3 independent experiments, *n*=55 and 60 for tau−/GVB− and tau?/GVB+ populations, respectively. ****p < 0.0001, Kolmogorov-Smirnov test for not normally distributed data

The only experimental model for GVBs in primary neurons to date relies on the induction of tau pathology by addition of exogenous PFFs to neurons expressing lentivirally transduced FTDtau^1^ [9]. Although we have previously shown that the addition of PFFs to untransduced neurons does not induce tau aggregation or GVB formation [9], this model did not allow the separation of tau aggregation from the addition of PFFs, which may be accompanied by endolysosomal damage [35–38] and thus contribute to GVB formation in the context of tau pathology. To further demonstrate that cytosolic pathological protein assemblies elicit GVB formation in the absence of exogenous tau PFFs, a seed-independent neuronal tau aggregation model was established by combination of FTDtau^1^ with another FTD mutation (P301L+S320F; FTDtau^1+2^). This double mutant tau variant was previously demonstrated to induce seed-independent aggregation in HEK293T cells [39], in mice in vivo [40] and ex vivo [41]. FTDtau^1+2^ lentiviral transduction at DIV 3 in primary mouse neurons (Fig. 1a) led to intracellular tau accumulations in ~80% of the neurons by DIV 18. These inclusions were MeOH-insoluble and positive for the MC1 antibody (Sup. Fig. 2a, Additional file 1), indicating the presence of insoluble pathological conformations of tau. In addition, the inclusions were positive for the p-tau epitope AT100 (Sup. Fig. 2b, Additional file 1). Insoluble pathological tau inclusions were not detected in FTDtau^1^ and untransduced control neurons (Sup. Fig. 2a, Additional file 1). In addition, the somata of 8–16% of the neurons bearing tau aggregates contained puncta positive for the established GVB marker pPERK [1] (Fig. 1b). To validate that the observed pPERK-positive structures are GVBs, co-staining with the GVB marker CK1δ was performed. This demonstrated strong co-localisation between the two markers as illustrated by the line intensity plot (Fig. 1c). Additional co-immunostaining showed that the CK1δ-positive structures are surrounded by a LIMP2-positive membrane (Fig. 1d). Together, these data demonstrate that the structures formed in this novel seed-independent model for tau pathology can be categorised as GVBs [10]. Importantly, these results show that intraneuronal tau aggregation elicits GVB formation independent of exogenous tau PFFs.

**Fig. 2:**
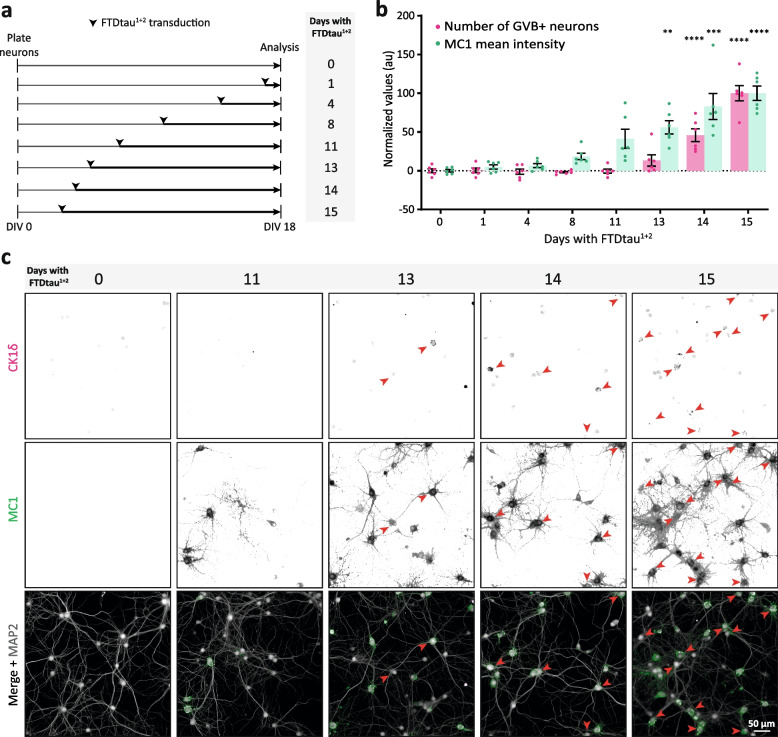
Pathological tau accumulation precedes GVB formation in the FTDtau^1+2^ model. **a** Schematic representation of experimental set-up to assess the temporal sequence of tau pathology and GVB formation. Plating and fixation day were the same as the regular protocol (Fig. 1a), whereas transduction with FTDtau^1+2^ was at different time points, resulting in different durations of exposure. **b** Quantification of the mean MC1 immunofluorescence intensity (green) and the number of GVB+ neurons (pink) per time point, relative to 15 days FTDtau^1+2^ exposure (100%). *N*=3 independent experiments, *n*=6 observations per time point, containing data from 5037–5775 neurons, were analysed. Data points represent the corrected mean value per well. Error bars represent the standard error of the mean (SEM). **p < 0.01; ***p < 0.001; ****p < 0.0001, compared to untransduced control (0 days), nested one-way ANOVA and Dunnett’s post hoc test. **c** Representative epifluorescence images for selected time points. Immunofluorescence staining was performed for the neuron-specific dendritic marker MAP2 (grey), MC1 (green) and CK1δ (magenta). Nuclei are visualised with DAPI (blue). Red arrowheads point to GVB+ neurons

To assess the causality between cytosolic tau pathology and GVB accumulation at the cellular level, quantitative single-cell analysis of MC1 tau intensity was performed in this newly developed tau/GVB model. FA-fixed samples were employed in order to include smaller pathological tau species that might be MeOH-soluble. Neurons containing GVBs (GVB+) were selected without prior knowledge of their tau pathology status (tau?/GVB+) and untransduced neurons were used as control (tau−/GVB−). Single-cell quantification of the somatic signal indicates an increase in average MC1 intensity in GVB+ neurons compared to control (Fig. 1e), demonstrating a strong connection between the presence of pathological tau and GVB accumulation. When comparing somatic pathological tau levels between GVB− and GVB+ neurons, no significant difference is found (Sup. Fig. 3, Additional file 1). This suggests that although the accumulation of cytosolic pathological protein assemblies is a prerequisite for eliciting GVB formation, additional factors appear to modulate the response. To investigate the sequence of events, the number of GVB+ neurons was assessed in FA-fixed neuronal cultures exposed to FTDtau^1+2^ for different durations ranging from 0 to 15 days (Fig. 2a). As quantified in Fig. 2b and illustrated in Fig. 2c, the appearance of MC1-positive inclusions and the presence of GVBs were progressive and increased with duration of FTDtau^1+2^ expression. Whereas intraneuronal MC1-positive inclusions were detected after 8 days of exposure, GVBs only started to appear after 13 days of FTDtau^1+2^ expression (Fig. 2b). These data demonstrate that the cytosolic accumulation of pathological tau assemblies precedes the occurrence of GVBs and support the conclusion that cytosolic aggregation of tau is a prerequisite for the induction of GVBs in this seed-independent model for tau pathology.

### GVB-bearing neurons in the hippocampus of tauopathy patients contain pathological tau

**Fig. 3:**
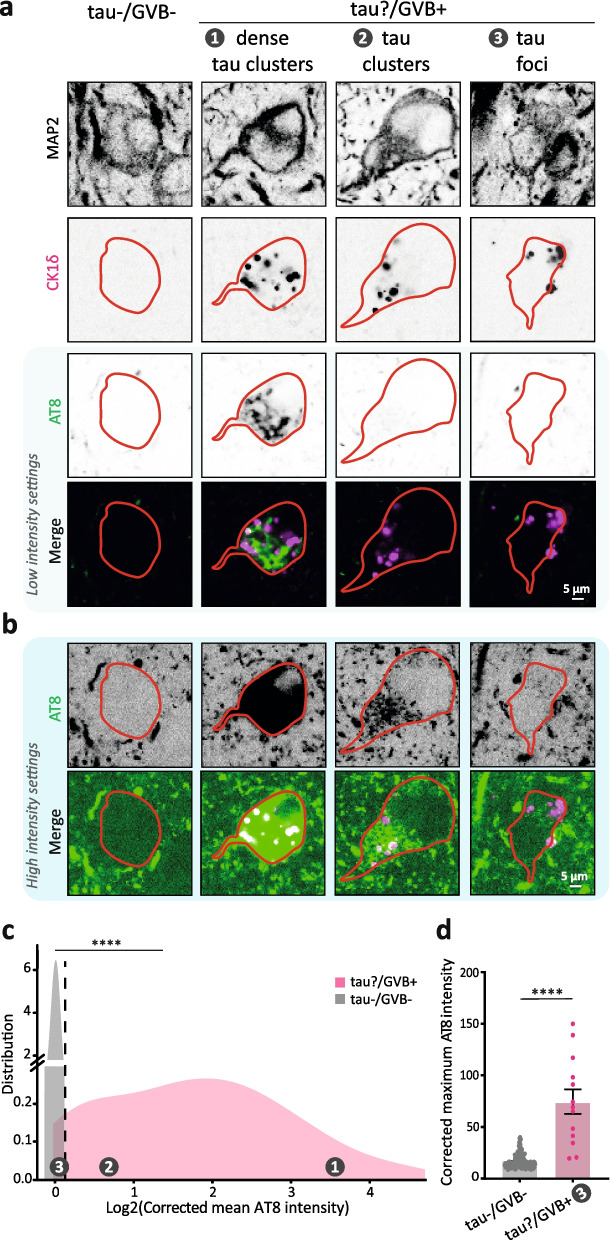
GVBs are associated with pathological tau accumulation in tauopathy brain. **a**–**d** Human post-mortem hippocampal tissue was analysed by immunofluorescence staining for the phosphorylated tau marker AT8, the GVB marker CK1δ and the neuron-specific dendritic marker MAP2. **a** Representative single plane confocal images of neurons without tau pathology or GVBs (tau−/GVB−) and neurons containing GVBs with (phenotype 1) and without (phenotypes 2 and 3) visible tau accumulations (tau?/GVB+). Separate channels are shown in greyscale, MAP2-based somatic mask is delineated in red and merged images show AT8 (green) and pPERK (magenta). **b** AT8 and merge from **a** at higher intensity to visualise low abundant tau accumulation in phenotypes 2 and 3. **c** Single-cell quantification of somatic pathological tau accumulation of tau−/GVB− (grey) and tau?/GVB+ (pink) neurons shown in a density plot as Log2-transformed corrected mean AT8 immunofluorescence intensity. *N*=4 patients, *n*=151 and 199 for tau−/GVB− and tau?/GVB+ populations, respectively. ****p < 0.0001, Kolmogorov-Smirnov test for not normally distributed data. **d** Corrected maximum AT8 intensity of GVB+ neurons with a Log2-transformed corrected mean tau intensity overlapping with that of GVB− neurons (left of the dotted line in **c**). Data points represent values from individual neurons. ****p < 0.0001, non-parametric Mann-Whitney *U* test. Error bars represent the standard error of the mean (SEM)

The reported occasional presence of GVBs in neurons lacking pathological tau immunopositivity in brains from tauopathy patients [3, 13–15, 17, 19–25] contrasts with the causal relation between tau aggregation and GVB formation found in mouse experimental models [9, 17, 18, 42–45] and the present study. This may indicate that GVBs can occur in the human tauopathy brain in the absence of pathological tau accumulation. Alternatively, these observations could be the result of limitations of conventional immunohistochemistry to detect low levels of pathological tau. To address this, we used immunofluorescence imaging for the early pathological p-tau epitope AT8 [46] to analyse hippocampal sections of tauopathy donors without regional α-syn comorbidity (Sup. Fig. 1a, b, Additional file 1). Similar to the experiment in Fig. 1e, GVB+ neurons were selected without prior knowledge of their tau pathology status (tau?/GVB+). Neurons in the same tissue section without GVBs or tau pathology (tau−/GVB−) were included as control. AT8 signal in these neurons was quantified in a ROI defined by the neuron-specific MAP2 (Fig. 3a, delineated in red). As previously reported [1, 4, 13–17], the majority of GVB+ neurons (75–85%) contain clearly visible tau pathology (tau+), often in the form of dense tau clusters (Fig. 3a, phenotype 1). Interestingly, intensity adjustment of the immunofluorescence images to detect low abundant AT8 positivity revealed 2 additional AT8 distributions (Fig. 3b): tau clusters that display lower intensity than those in phenotype 1 (phenotype 2) and tau foci, where local low-intensity AT8 accumulations are present (phenotype 3). The specificity of the signal was supported by a staining control in which the AT8 antibody was omitted (Sup. Fig. 4a, Additional file 1). In line with these observations, quantification of the mean AT8 intensity per neuron (Fig. 3c) showed increased levels of AT8 signal in the great majority (93.5%) of GVB+ neurons compared to tau−/GVB− neurons. The AT8 signal in the small, local tau accumulations in phenotype 3 is averaged out when quantifying mean values. Therefore, the minor fraction of GVB+ neurons that did not show increased AT8 mean intensity (left of dashed line in Fig. 3c) was further analysed by determining the maximum AT8 signal intensity per neuron (Fig. 3d). This analysis demonstrated increased maximum AT8 signal in these GVB+ neurons compared to GVB−/tau− neurons. We therefore conclude that the occurrence of GVBs in tauopathy hippocampus is inseparably associated with the presence of pathological tau.

### A-syn pathology induces GVB formation

**Fig. 4:**
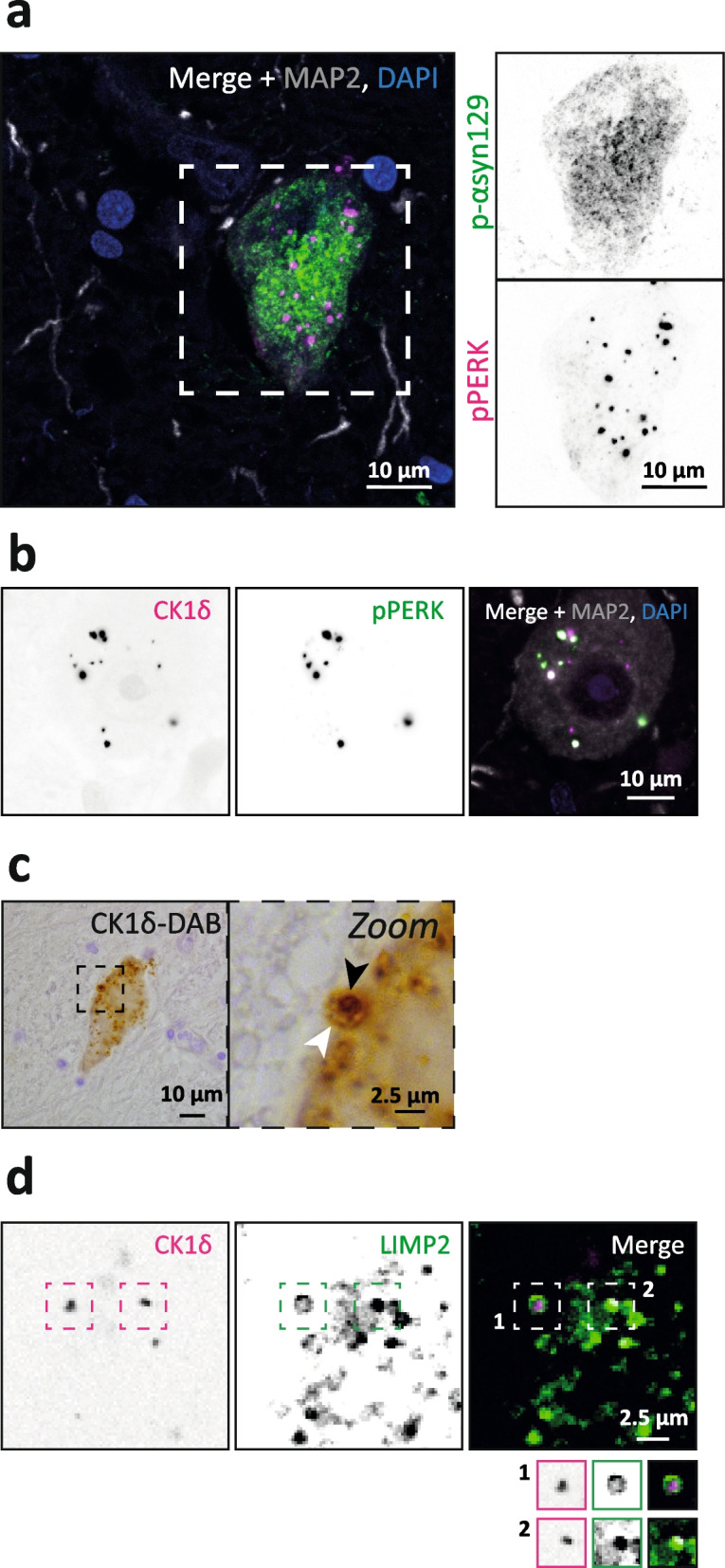
Identification of GVBs in human PD *substantia nigra*. **a** Representative confocal images of a GVB+ neuron in the PD SN showing the GVB marker pPERK (magenta), the p-αsyn129 marker for phosphorylated α-syn (green) and the neuron-specific dendrite marker MAP2 (grey). **b** Representative confocal images of a GVB+ neuron in the PD SN showing co-localisation of the canonical GVB marker CK1δ (magenta) and the additional GVB core marker pPERK (green). The neuron-specific marker MAP2 is in grey. Nuclei are visualised with DAPI (blue). **c** Immunohistochemical DAB staining of CK1δ and haematoxylin to show the typical GVB morphology. The black arrowhead in the zoom shows the dense GVB core and the white arrowhead points to the GVB membrane. **d** Representative confocal images of a GVB+ neuron in the PD SN showing the GVB marker CK1δ (magenta) and the lysosomal membrane marker LIMP2 (green). Insets for two GVBs are separately shown for better visualisation of the spatial relation between the GVB membrane marker and the core

Our data demonstrate a causal relationship between intraneuronal tau pathological assemblies and GVBs in primary mouse neurons (Figs. 1 and 2) and a tight association of GVBs with pathological tau in human tauopathy brain (Fig. 3). The presence of GVB-like structures has also been reported in association to α-syn pathologies, including PD [11, 26–30]. To conclusively determine whether GVBs are found in association with α-syn pathology, we studied if GVBs found in the SN of brain donors diagnosed with PD adhere to the proposed criteria [10]. Immunofluorescence co-staining for the phosphorylated α-syn epitope p-αsyn129 and the GVB marker pPERK demonstrated the presence of pPERK-positive puncta in a subset of neurons with α-syn pathology (Fig. 4a). Co-localisation of the pPERK puncta with CK1δ was observed for most structures (Fig. 4b). In addition, CK1δ immunostaining and DAB labeling demonstrated that the GVB dense core and vacuole were surrounded by a membrane (Fig. 4c). Immunofluorescence co-staining for CK1δ and LIMP2 often showed LIMP2 signal surrounding or overlapping with CK1δ-positive puncta (Fig. 4d), demonstrating the lysosomal nature of the GVB membrane. Thus, GVBs in PD SN have the typical GVB morphology. GVB presence and identity was validated in all PD cases included in this study (Sup. Figs. 5 and 6, Additional file 1). Interestingly, it was observed that GVBs in LB-containing neurons often localised in a close perimeter around the inclusion body (Sup. Fig. 5a, Additional file 1), whereas GVBs in neurons with more diffusely distributed α-syn pathology were more scattered throughout the neuronal soma (Fig. 4a and Sup. Fig. 5b, Additional file 1), similar to the tau-associated GVBs in AD brain.

**Fig. 5:**
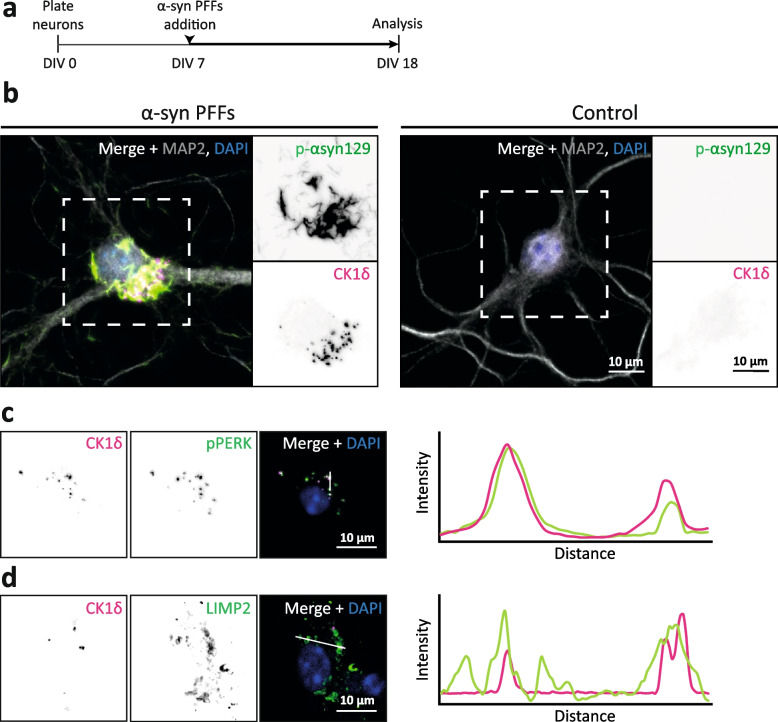
GVBs are found in association with pathological α-synuclein accumulation in the seeded model. **a** Schematic representation of the protocol to induce intraneuronal α-syn pathology in primary mouse neurons. WT neurons were plated at DIV 0. At DIV 7, α-syn PFFs were added to the culture medium. Cells were fixed and analysed by immunofluorescent staining for pathological α-syn and GVBs at DIV 18. **b** Representative confocal images showing an α-syn+/GVB+ (left) and an α-syn−/GVB− control neuron (right) to which the seed vehicle (PBS) was added. Immunofluorescence staining was performed for the neuron-specific dendritic marker MAP2 (grey), phosphorylated α-syn (p-αsyn129, green) and GVB marker CK1δ (magenta). Separate channels are shown in greyscale. **c**, **d** Representative confocal images of GVB+ neurons in the α-syn model. Co-immunofluorescence staining was performed for the canonical GVB marker CK1δ (magenta) and the additional GVB core marker pPERK (**c**) or the GVB membrane marker LIMP2 (**d**) (green). Nuclei are visualised by DAPI (blue). Separate channels are shown in greyscale and in colour in the merge. A line intensity profile along the white bar indicated in the merge shows CK1δ signal overlap with pPERK and being surrounded by LIMP2

To determine whether there is a causal connection between α-syn aggregates and GVB formation, a primary neuron model for α-syn pathology was employed [47]. Here, aggregation of endogenous α-syn is achieved by addition of α-syn PFFs to the media of WT untransduced neurons (Fig. 5a). In the presence of α-syn PFFs, MeOH-insoluble p-αsyn129-positive α-syn accumulations were prominently observed in neurites and to a lesser extent in neuronal somata of ~20–50% of the neurons (α-syn+, Sup. Fig. 7a, Additional file 1). CK1δ-positive puncta were observed in ~1–10% of these neurons and were often accompanied by the presence of α-syn pathological assemblies in the soma (Fig. 5b and Sup. Fig. 7b, Additional file 1). Neurons in the vehicle control condition did not show aggregated α-syn or CK1δ-positive puncta. To investigate whether the CK1δ-positive puncta correspond to GVBs, further validation with GVB markers was performed. Line intensity plots show strong co-localisation of CK1δ-positive puncta with the GVB core marker pPERK (Fig. 5c). In addition, CK1δ puncta are enclosed by a LIMP2-positive membrane (Fig. 5d). Together, these data demonstrate that GVBs are found in association with cytosolic pathological α-syn assemblies in primary neurons and in the brain of PD patients.

### GVBs induced by α-syn pathology are independent of pathological tau accumulation

An alternative explanation to the accumulation of GVBs in neurons with α-syn pathology could be the presence of tau pathological species in the same neuron. Double immunohistochemistry for CK1δ and p-αsyn129 showed regional co-occurrence of pathological α-syn and GVBs in PD SN tissue, whereas p-αsyn129 signal was not present in the hippocampal sections of the tauopathy patient cohort (Sup. Fig. 1b, d, Additional file 1). In addition, double immunohistochemistry using AT8 and CK1δ demonstrated the absence of p-tau in the analysed PD SN tissue, in contrast to the high pathological tau content present in hippocampal sections derived from tauopathy brain (Sup. Fig. 1a, c, Additional file 1). This demonstrates the absence of tau comorbidity in the PD SN sections used in this study, suggesting that GVBs are induced by α-syn pathology in the absence of pathological tau accumulation.

**Fig. 6:**
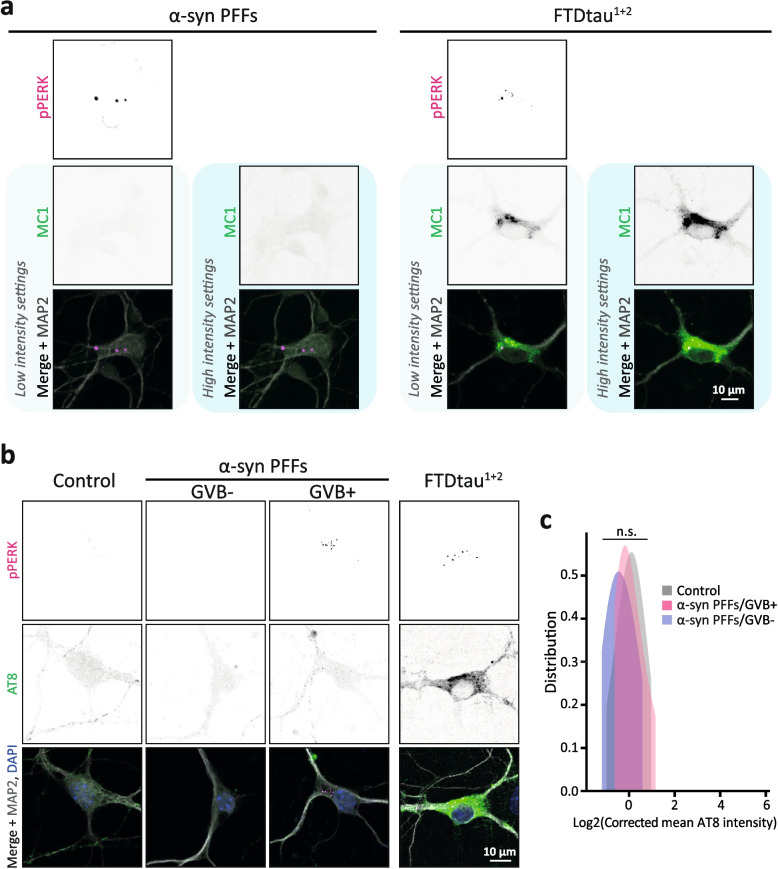
Pathological α-synuclein accumulation induces GVBs in the absence of pathological tau. **a** Representative confocal images of immunofluorescence staining for MC1 (green) and pPERK (magenta). Separate channels are in greyscale and MAP2 is shown in grey in the merged images. FTDtau^1+2^-transduced neurons were used to set MC1 imaging settings for the detection of pathological tau and are shown as a reference. MC1 and merged images are also shown at increased intensity to visualise low abundant pathological tau. **b**, **c** Single-cell quantification of pathological tau load in GVB− and GVB+ neurons in the α-syn pathology model. **b** Representative confocal images of primary mouse neurons treated with α-syn PFFs to induce α-syn pathology or the seed vehicle as control. Immunofluorescence staining was performed for AT8 (green) and pPERK (magenta). Separate channels are shown in greyscale and merged images show neurons in grey and nuclei in blue. FTDtau^1+2^-transduced neurons were used to set AT8 imaging settings for the detection of pathological tau and are shown as a reference. **c** Single-cell quantification of somatic tau load in GVB− (blue) and GVB+ (pink) neurons in the α-syn model represented as the Log2-transformed corrected mean AT8 intensity. Vehicle-treated control neurons are shown in grey. *N*=3 independent experiments, *n*=64, 17 and 123 for control, α-syn PFFs/GVB+ and α-syn PFFs/GVB− populations, respectively. n.s. not significant, nested one-way ANOVA with Tukey’s post hoc test

The newly developed α-syn/GVB model facilitated single-cell assessment of pathological tau in GVB+ neurons in the context of α-syn pathology. This demonstrated that GVB+ neurons in this experimental paradigm are not immunopositive for the pathological tau antibody MC1 (Fig. 6a). Low level of AT8 positivity was observed in neurons in all groups, including vehicle-treated neurons (Fig. 6b). This is commonly reported in mouse neurons in the absence of pathology [48] and consistent with results from human tissue of cognitively normal individuals [49]. The observed signal was very distinct from the abundant presence of MC1- and AT8-positive tau accumulations in the spontaneous FTDtau^1+2^ model used as positive control (Fig. 6a, b). To exclude the presence of low abundant pathological tau species in GVB+ neurons in the α-syn pathology paradigm, the same quantitative analysis employed in the tauopathy context (Figs. 1 and 3) was performed for the AT8 signal. This demonstrated that the mean AT8 intensity levels of GVB− and GVB+ neurons in the α-syn pathology model were not different between them or from control neurons (Fig. 6c). These data show that α-syn aggregation results in GVB formation independent of cytosolic pathological tau accumulation.

### Similar p-tau immunoreactivity signature of GVBs induced by tau and α-syn pathology

**Fig. 7:**
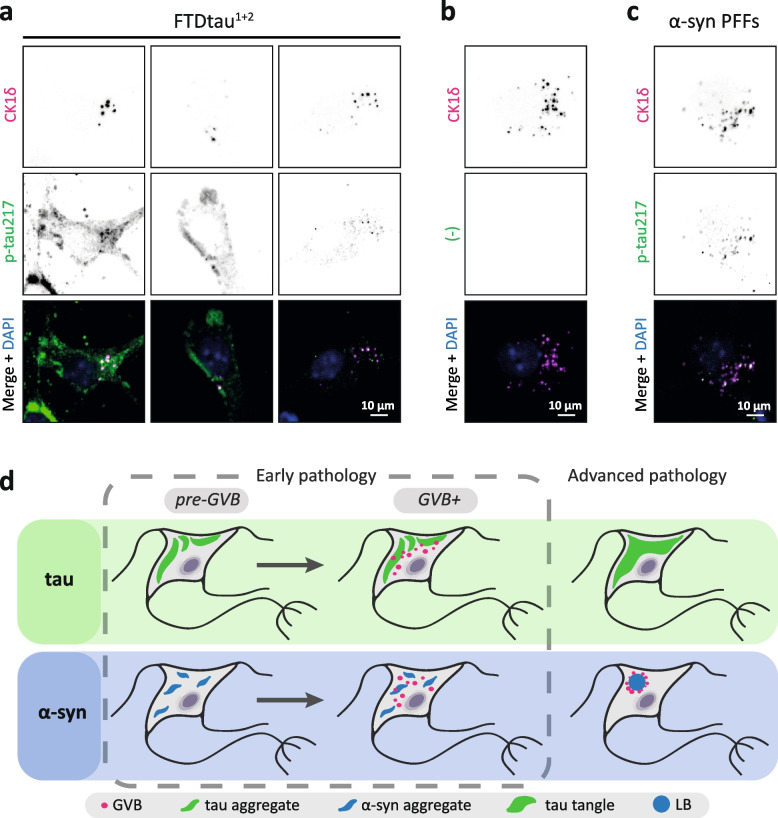
GVBs independently induced by tau and α-synuclein pathology are p-tau217-positive. **a**–**c** Immunofluorescence co-staining for CK1δ (magenta) and p-tau217 (green) in our experimental GVB models. Separate channels are shown in greyscale and nuclei are visualised with DAPI (blue) in the merged images. **a** Representative confocal images of GVB+ neurons in the FTDtau^1+2^ model show a strong overlap between CK1δ puncta and p-tau217 in addition to the cytosolic tau pathology also marked by the phospho-tau epitope. Several examples with varying levels of somatic pathology are shown for better visualisation **b** Representative confocal images of a GVB+ neuron in a staining control condition for which p-tau217 primary antibody was omitted. **c** Representative confocal images of a GVB+ neuron in the seeded α-syn pathology model show a similar overlap between GVB markers as observed for the tau/GVB model in the absence of signal for somatic tau pathology. **d** Schematic summary: GVBs are inseparably associated with pathological protein accumulations and can be independently induced by cytosolic tau and α-syn pathological protein assemblies. Neurons with advanced tau pathology in the human brain are devoid of GVBs. In contrast, GVBs are observed in neurons with advanced α-syn pathology. Our data indicate that GVBs are a generalised lysosomal response to disturbed proteostasis

Having demonstrated that GVBs are induced by α-syn aggregation in the absence of pathological tau (Fig. 6), the experimental GVB models introduced in this study provide a unique opportunity to investigate shared or distinct GVB markers depending on the GVB-inducing pathology. Here, we specifically asked the question whether p-tau positivity in GVBs is linked to the presence of cytosolic tau pathology. Immunofluorescence co-staining for pPERK and the pathological tau markers MC1 (Figs. 1b and 7a and Sup. Fig. 3a), AT100 (Sup. Fig. 2b) and AT8 (Fig. 7b) in the tau/GVB model showed occasional overlapping signal, as was previously reported in the seeded tau/GVB model [9]. These results are in line with our observations of GVBs in tissue from tauopathy patients (Fig. 3a) and in agreement with reported p-tau immunoreactivity of GVBs in the human brain (reviewed in [2]). Immunofluorescence co-staining for p-tau217 and CK1δ in the tau/GVB model showed that in addition to labeling tau pathology, p-tau217 strongly co-localises with CK1δ-positive puncta (Fig. 7a) whereas no signal was found in the absence of p-tau217 primary antibody (Fig. 7b). These results are in agreement with the observations in human brain material from AD patients [50]. A similar co-staining in the tauopathy patient cohort showed p-tau217 immunopositivity of the tau pathology. However, in contrast with the previous report that used a different, non-commercial antibody [50], no p-tau217 signal overlapping with CK1δ puncta was found when compared to the staining control (Sup. Fig. 8a and b, Additional file 1). Interestingly, in SN brain material from PD patients, p-tau217 signal was detected in a punctate pattern and overlapping with ~60% of CK1δ puncta (Sup. Fig. 8c, Additional file 1) when compared to the negative staining control (Sup. Fig. 8d, Additional file 1), indicating that GVBs in PD brain are immunopositive for p-tau217. We conclude that the absence of p-tau217 immunopositivity in GVBs in tauopathy brain is not due to incompatibility of the antibody with GVBs in human brain, but most likely caused by GVB target distraction of the antibody by the abundant tau pathology, which is not present in PD. This could not be solved by increasing the antibody concentration (data not shown).

Finally, the α-syn/GVB model was studied using p-tau217/CK1δ double immunofluorescence. As expected from the absence of cytosolic pathological tau accumulations (Fig. 6) and in accordance with observations in the human PD brain (Sup. Fig. 8c, Additional file 1), neurons in the α-syn/GVB model did not show aggregate-like p-tau217 accumulation (Fig. 7c). Similar to GVBs in the tauopathy context, GVBs in this model showed inconsistent overlapping signal with pathological tau markers MC1 (Fig. 7a) and AT8 (Fig. 7b). However, a strong co-localisation between p-tau217 and CK1δ puncta comparable to the tau/GVB model was observed (Fig. 7c). We conclude that the p-tau immunoreactivity signature is similar for GVBs induced in response to cytosolic pathological assemblies of either α-syn or tau.

## Discussion

Here, we studied the connection between pathological protein assemblies and GVB formation. Employing patient brain material as well as novel neuronal GVB models, we demonstrated that GVBs in the context of tauopathy are inseparably associated with cytosolic pathological tau accumulations and that the aggregation of tau precedes GVB formation, further strengthening the causal link between these two events. In addition, we showed that GVBs are found in association with α-syn accumulation in the human brain and that they form as a result of α-syn pathology in the first α-syn/GVB primary neuron model to date, where we demonstrate that this process is independent of cytosolic pathological tau accumulation. Interestingly, we show that GVBs induced by either cytosolic pathological tau or α-syn accumulations display a similar p-tau immunoreactivity profile.

In the tauopathy brain, GVBs [17, 19–23] and putative GVBs [3, 13–15, 24, 25] are occasionally observed in neurons without apparent tau pathology. Based on semiquantitative visual assessment, previous studies have reported a range of 5–25% [13–15, 17, 20] up to even 80% [25] of GVB+ neurons without pathological tau. In the current study, we performed systematic analysis of pathological tau at the single-cell level. Using this quantitative method, we show that GVB+ neurons have increased mean AT8 intensity values, indicating the presence of pathological tau in these neurons. However, visual assessment would have resulted in a proportion of GVB+ neurons without tau pathology of approximately 15–25%, similar to what has been reported in literature. These results highlight the importance of performing quantitative analysis since visual assessment of the neuronal status as containing or not pathological tau species is biased towards stronger pathology. Despite the loss of p-tau epitopes during the post-mortem interval [51, 52], our quantitative analysis of pathological tau levels in hippocampal sections from tauopathy brain shows that in only 1.8% of the analysed GVB+ neurons increased pathological tau levels were not detected. Moreover, we conclude from these data that low abundant or early pathological tau species can elicit GVB formation as observed in the case of tau foci, in which the pathology is barely detectable. This is in agreement with previous studies that identify GVBs as an early pathological hallmark of tauopathies [1–4].

GVBs were also observed in association with α-syn pathology in the brain of PD patients. Although GVBs are not found in tangle-bearing neurons in brains of tauopathy patients [2], we identified GVBs in neurons with advanced stages of α-syn pathology (LBs). Interestingly, GVBs in LB-containing neurons were localised in a close perimeter around the inclusion body, in contrast to the more scattered distribution in neurons with early α-syn or tau pathology. This phenomenon has been observed before for sialic acid-positive structures [30], which could correspond to GVBs since pPERK-, phosphorylated TAR DNA-binding protein 43 (pTDP-43)- and LAMP1-positive puncta in the context of AD are shown to be highly positive for sialic acid in the same study. It is tempting to speculate that this could be related to the recruitment of cytoskeletal elements to the outer edge of LBs [53], but this requires further study.

Due to the high occurrence of comorbidities in the human brain, GVB+ neurons occasionally reported in the absence of pathological tau in literature may be accompanied by pathological conformations of other proteins. The use of experimental disease models such as the ones we have developed here offers the possibility to study pathologies independently, address causality and assess disease-modifying approaches. In view of the potential lysosomal damage induced by PFFs [35–38], we now established a seed-independent tau pathology model that conclusively shows that GVBs form as a result of cytosolic aggregation in the absence of exogenous seeds. A limitation of this model is that it requires lentiviral transduction, which could potentially introduce artefacts. However, previous work from our lab showed that lentiviral transduction of single mutant FTDtau^1^ does not result in GVBs [9], demonstrating that this experimental intervention is not a confounding factor in the model. The seed-independent tau/GVB model allowed single-cell quantification of pathological tau without the limitation of post-mortem artefacts and showed tau pathology in every GVB+ neuron. This spontaneous tau/GVB model offers a more reproducible and robust outcome in terms of tau pathology and GVB load by minimising the high variability that the PFF component introduces in relation to batch-to-batch differences in seed production, sonication and cellular uptake. These characteristics make it a simple and suitable model to perform higher throughput experiments aiming to characterise molecular mechanisms underlying GVB formation and their significance in protein pathologies. Using a previously described model for seeding with α-syn PFFs [47], we demonstrated for the first time a causal connection between cytosolic pathological α-syn assemblies and GVBs. The α-syn/GVB model employs endogenous α-syn and does not involve lentiviral transduction, hence providing additional evidence that lentiviral transduction per se is not necessary for the induction of GVBs.

The evidence we present here demonstrates that GVBs form in response to pathological tau and α-syn. GVB-triggering pathological protein accumulations are intracellular and localised in the cytosol. In accordance, no association with GVBs has been found for nuclear huntingtin inclusions [54] and extracellular pathological prion protein deposits [55] or amyloid beta plaques [3, 17, 18]. Tau and α-syn pathologies are found in different neuronal subtypes and brain regions. This is in line with our previous report that although GVB formation selectively occurs in neurons, they are not dependent on a specific neuronal subtype [9]. Similarly, in the present study we established the α-syn/GVB model in cortical mouse neurons that recapitulates the observations made in dopaminergic neurons in the SN of human PD brain. Tau and α-syn are very different in protein sequence and structure. Additionally, increased insight in structures of tau filaments [56] suggests that the seed-induced FTDtau^1^ and the spontaneous FTDtau^1+2^ aggregation are structurally different, yet both induce GVBs. Also, in the human brain GVBs are observed in different tauopathies independent of the tau isoform in the inclusions or the presence of a *MAPT* mutation (reviewed in [10]). Therefore, our experimental data strengthen the conclusion that GVBs do not require a specific protein structure.

Putative GVBs have also been associated with other proteinopathies in the human brain [10]. In two of these studies, the absence of regional tau comorbidity was confirmed by immunostaining for p-tau. The first involves pathological protein assemblies of fused-in-sarcoma protein (FUS) in one patient with an ALS-like phenotype and FUS inclusions [3]. The most prominent evidence for GVB formation in the absence of tau or α-syn pathological assemblies is attributed to dipeptide repeat (DPR) inclusions in cases of frontotemporal lobar degeneration or amyotrophic lateral sclerosis caused by a *C9ORF72* repeat expansion (C9) [57, 58]. Recently, a strong association was shown between GVBs and DPR inclusions in the absence of pathological TDP-43 and tau assemblies [59]. In this study, DPR-associated GVBs were found in the cerebellum, a brain region that is generally devoid of tau pathology. These data support the formation of GVBs by DPR pathology; however, the potential causal relation between DPR inclusions and GVBs should be addressed in future studies using experimental models for DPR aggregation.

Previous work from our group showed that GVBs are active lysosomes and it may be expected that in the presence of tau pathology there is a high demand for degradation of tau species in GVBs. Despite the occasional detection of (phosphorylated) tau in GVBs (reviewed in [2]), the lack of fluorescently tagged tau accumulation in experimental GVBs [9] may indicate that this process is highly efficient. A recent study has identified a consistent association of p-tau217 with GVBs in the brain of AD patients in contrast to the only occasional positivity for other phosphorylated tau (p-tau) epitopes: p-tau181, p-tau231, p-tau202/205 and p-tau369/404 [50]. P-tau217 is emerging as a robust and selective cerebrospinal fluid (CSF) [60, 61] and plasma [62] biomarker for tau pathology in AD and it was hypothesised that its selective accumulation in GVBs could provide the mechanistic explanation for increased presence in AD CSF and plasma [50]. Since p-tau217 CSF and plasma levels are not increased in PD patients [61, 62], it is paradoxical that GVBs are also found in the context of α-syn pathology and could suggest variation in GVBs induced by different protein pathologies. In the current study, we demonstrate p-tau217 immunopositivity in GVBs in both the tau/GVB model and the α-syn/GVB model, implying that p-tau217 positivity in GVBs is not directly related to the presence of cytosolic tau pathology. Importantly, the immunopositivity for p-tau217 does not conclusively demonstrate that p-tau217 is present in GVBs. In fact, as indicated in the introduction, the only cargo that has been shown to selectively accumulate in GVBs without the use of immunodetection is CK1δ [9]. Phosphorylated epitopes are abundantly observed in GVBs, however, without consistent detection of their non-phosphorylated-specific counterparts, as is the case for tau [2]. Further investigation employing tau-deficient neurons is required to validate the presence of this p-tau epitope in GVBs and its specificity. The potential recruitment of p-tau217 to GVBs independent of the inducing protein pathology could suggest a potential role for tau in GVB formation apart from its presence in cytosolic pathological protein assemblies. It is tempting to speculate about a role for phosphorylation of tau at position 217 as a stress response to proteostatic disturbance, but that requires further investigation. Wennström et al. [50] hypothesised a potential role for GVBs as secretory vesicles that result in release of p-tau217, ultimately reflected in body fluids. Our results suggest that the discriminative capacity of CSF and plasma p-tau217 levels for AD and PD is not connected to differential presence of p-tau217 in GVBs, although it cannot be excluded that distinct secretion rates or exposure of the affected brain areas to the CSF and blood stream underlie the differential p-tau217 levels observed in body fluids in these proteinopathies.

A major strength of this study is the combination of data obtained in experimental models and observations in the human brain. We exploited the unique opportunity our models provide to identify causal relations between cytosolic pathological protein assemblies and GVBs and confirmed these observations in the human brain. The number of human cases analysed in our study is relatively low, but these are largely used as qualitative validation for our data from the novel experimental GVB models and provide consistent results. In addition, due to less efficient pathology induction in the seeded α-syn/GVB model in comparison to the spontaneous tau/GVB model, the number of GVB+ neurons is lower for the α-syn model. Spontaneous α-syn pathology has been reported in mouse slices by overexpression of human WT or A53T α-syn [41]; however, this manipulation did not result in spontaneous α-syn aggregation in our primary mouse cultures (data not shown). Future efforts should be directed to the development of more efficient α-syn/GVB models to achieve a higher throughput. The limited number of markers employed for the assessment of pathological tau in the α-syn/GVB model could be considered as another limitation of this study. Quantification of a p-tau marker (AT8) for early tau pathology, as proposed by observations in the human brain [46], in FA-fixed samples was performed to optimally detect early pathological tau species in our α-syn/GVB model.

Our study identified GVB formation as a more generalised response to cytosolic pathological protein assemblies (see schematic in Fig. 7d). It had been observed previously that not all neurons containing cytosolic accumulation of pathological tau develop GVBs in the human brain [1, 12, 22]. This is reproduced in the seed-dependent tau/GVB model [9] as well as the seed-independent tau/GVB model we present in this study. Our single-cell quantitative analysis of tau+/GVB− and tau+/GVB+ shows that the somatic tau pathology load does not differ between GVB+ and GVB− neurons, indicating that the extent of cytosolic pathology is not a direct determinant of GVB formation. This suggests that neurons may exhibit differential susceptibility to cytosolic protein aggregates, for example by differences in proteolytic capacity. Alternatively, this could involve a factor downstream of pathological protein accumulation, e.g. the differential activation and recruitment of proteins mechanistically involved in GVB assembly. It is possible that all neurons containing cytosolic protein aggregates undergo a transient GVB+ stage that could potentially dictate their transition towards a degenerative state or alleviation of pathology followed by GVB loss (see [10] for a more in depth discussion). This would be in agreement with the observation that tangle-bearing neurons are not found in association with GVBs [2]. In this respect, it is interesting to note that GVBs are present in LB-positive neurons. This could suggest that a different mechanism is involved in tau- and α-syn-induced GVB formation, or could indicate that neurons with end-stage tau or α-syn pathology represent a different neuronal state. The novel GVB models we present here will greatly facilitate more detailed study of the mechanisms and implications of GVB formation.

Understanding the neuronal responses to protein aggregates will lead to identifying key molecular mechanisms that could be used to discover therapeutic targets to combat neurodegenerative proteinopathies. GVBs may embody a converging early lysosomal stress response to cytosolic pathological protein assemblies that is neuron-specific. Known lysosomal stress responses involve either transcriptional activation that enhances overall lysosomal biogenesis mediated by the activation of the transcription factor EB (TFEB) (for review see [63]) or a trafficking response resulting in a relocalisation of lysosomes to the perinuclear area (for review, see [64]), which is in line with the near exclusive somatic position of GVBs, both in the context of tau and α-syn pathology. The GVB models we describe in this paper provide novel tools to investigate whether one or both of these responses are involved in GVB formation. Future research directed at the study of shared features downstream of tau and α-syn aggregation will shed light on the role of these enigmatic structures in neurodegenerative disorders and decipher whether they are detrimental or beneficial in the disease process. The identification of GVBs as common early player in a broad spectrum of neurodegenerative disorders makes them an attractive target for disease-modifying therapeutic strategies.

## Conclusions

Our results demonstrate that pathological protein assemblies are required for GVB formation and that GVBs are independently induced by cytosolic tau and α-syn pathological accumulation. Our data identify the emergence of GVBs as a generalised gain-of-function response to cytosolic protein pathology.

## Supplementary Information

**Additional file 1: Supplementary Figure 1.** Neuropathology of the analysed GVB+ human brain tissue. **Supplementary Figure 2.** FTDtau^1+2^ induces intracellular accumulation of phosphorylated, insoluble tau. **Supplementary Figure 3.** Pathological tau levels are not different between GVB- and GVB+ neurons in tau/GVB model. **Supplementary Figure 4.** Negative staining control for Fig. 3. **Supplementary Figure 5.** Characterisation of GVBs in human PD *substantia nigra*. **Supplementary Figure 6.** Validation of GVB identity in human PD *substantia nigra*. **Supplementary Figure 7.** Characterisation of the pathology induced in the α-synuclein seeded model. **Supplementary Figure 8.** P-tau217 immunofluorescence in human brain tissue. **Supplementary Table 1.** Overview of independent experiments or patients and analysed cells per figure.

## Data Availability

The data that support the findings of this study are available from the corresponding author upon reasonable request.

## Abbreviations

AD: Alzheimer’s disease
C9: C9ORF72 repeat expansion
CSF: Cerebrospinal fluid
DAB: 3,3′Diaminobenzidine
DAPI: 4′,6-Diamidino-2-phenylindole
DPR: Dipeptide repeat
FA: Formaldehyde
FTD: Frontotemporal dementia
FTDtau^1^: Tau P301L
FTDtau^1+2^: Tau P301L, S320F
FUS: Fused-in-sarcoma protein
GVBs: Granulovacuolar degeneration bodies
HRP: Horseradish peroxidase
LB: Lewy body
LIMP2: Lysosomal integral membrane protein 2
MAP 2: Microtubule-associated protein-2
MeOH: Methanol
MSA: Multiple system atrophy
NFTs: Neurofibrillary tangles
O/N: Overnight
PBS: Phosphate saline buffer
PD: Parkinson’s disease
PFFs: Pre-formed fibrils
pPERK: Phosphorylated protein kinase R (PKR)-like endoplasmic reticulum kinase
p-tau: Phosphorylated tau
pTDP-43: Phosphorylated TAR DNA-binding protein 43
ROI: Region of interest
RT: Room temperature
SN: *Substantia nigra*
TDP-43: TAR DNA-binding protein 43
TFEB: Transcription factor EB
WT: Wild type
α-syn: α-synuclein
