# Supplementary material for: Granulovacuolar degeneration bodies are independently induced by tau and α-synuclein pathology
Source: Alzheimers Res Ther. 2022 Dec 14;14:187. doi: 10.1186/s13195-022-01128-y (PMC9749177; doi:10.1186/s13195-022-01128-y)
Supplement: Supplementary file 1 — Additional file 1: Supplementary Figure 1. Neuropathology of the analysed GVB+ human brain tissue. Supplementary Figure 2. FTDtau1+2 induces intracellular accumulation of phosphorylated, insoluble tau. Supplementary Figure 3. Pathological tau levels are not different between GVB- and GVB+ neurons in tau/GVB model. Supplementary Figure 4. Negative staining control for Fig. 3. Supplementary Figure 5. Characterisation of GVBs in human PD substantia nigra. Supplementary Figure 6. Validation of GVB identity in human PD substantia nigra. Supplementary Figure 7. Characterisation of the pathology induced in the α-synuclein seeded model. Supplementary Figure 8. P-tau217 immunofluorescence in human brain tissue. Supplementary Table 1. Overview of independent experiments or patients and analysed cells per figure. [file 13195_2022_1128_MOESM1_ESM.pdf]

Supplementary information

Granulovacuolar degeneration bodies are independently induced by tau and α-synuclein pathology

Marta Jorge-Oliva[1], Jasper F. M. Smits[1], Vera I. Wiersma[1, 2], Jeroen J. M. Hoozemans[3, 4], Wiep Scheper[1, 2, 4*]

[1] *Dept. of Functional Genomics, Center for Neurogenomics and Cognitive Research, Vrije Universiteit (VU), De Boelelaan 1085, 1081 HV Amsterdam, the Netherlands*

[2] *Dept. of Human Genetics, Amsterdam UMC location Vrije Universiteit, De Boelelaan 1117, 1081 HV Amsterdam, the Netherlands*

[3] *Dept. of Pathology, Amsterdam UMC location Vrije Universiteit, De Boelelaan 1117, 1081 HV Amsterdam, the Netherlands*

[4] *Amsterdam Neuroscience, Neurodegeneration, Amsterdam, the Netherlands*

*Correspondence to

W. Scheper

VU Faculty of Science

Center for Neurogenomics and Cognitive Research

Department of Functional Genomics

De Boelelaan 1085

1081 HV Amsterdam

The Netherlands

E-mail: w.scheper@amsterdamumc.nl

Phone: +31-20-5982771

Supplementary Figure 1

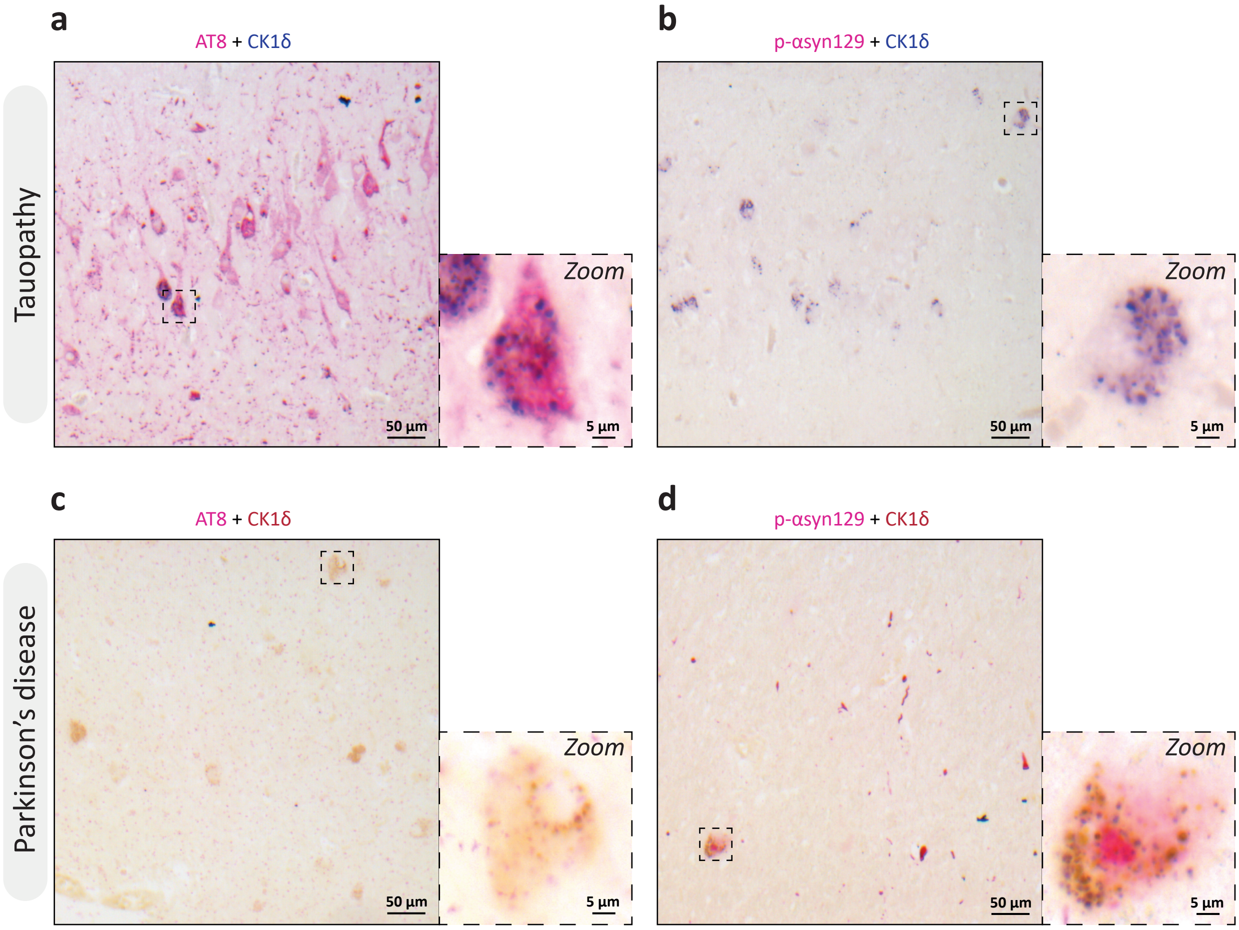

Supplementary Figure 1 Neuropathology of the analysed GVB+ human brain tissue. **a-d** Representative examples of double immunohistochemical staining for AT8 in liquid permanent red (LPR) in combination with CK1δ in VIP (a) or CK1δ in DAB (c) and p-αsyn129 staining in LPR in combination with CK1δ in VIP (b) or CK1δ in DAB (d). **a,b** Section of tauopathy hippocampus showing abundant GVB+ neurons, which are associated to pathological tau accumulations (a). GVB+ neurons in this material are devoid of α-syn pathological accumulations (b). **c,d** Section of PD SN showing that GVB+ neurons do not show hyperphosphorylated tau (c), but are found in neurons with α-syn pathological accumulations (d). Representative examples of GVB+ neurons are shown in the zooms.

Supplementary Figure 2

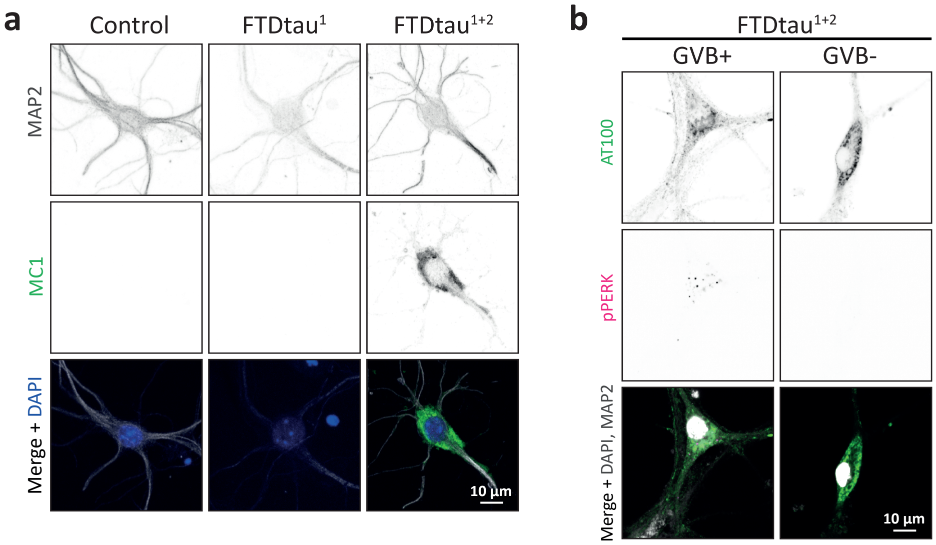

Supplementary Figure 2 FTDtau^{1+2} induces intracellular accumulation of phosphorylated, insoluble tau. **a** Representative confocal images of MeOH-fixed neurons following the same experimental setup as in Fig. 1a. Neurons transduced with FTDtau^{1+2}, FTDtau^{1} and untransduced control were included. Immunofluorescence staining was performed for MAP2 (grey) and MC1 (green). Nuclei are visualised by DAPI (blue). Separate channels are shown in greyscale. **b** Representative confocal images of GVB+ (left) and GVB- (right) neurons in the FTDtau^{1+2} model. Immunofluorescence staining was performed for MAP2 (grey), pPERK (magenta) and the tau phosphorylation marker AT100 (green). Nuclei are visualised by DAPI (grey). Separate channels are shown in greyscale. Single focal planes were acquired.

Supplementary Figure 3

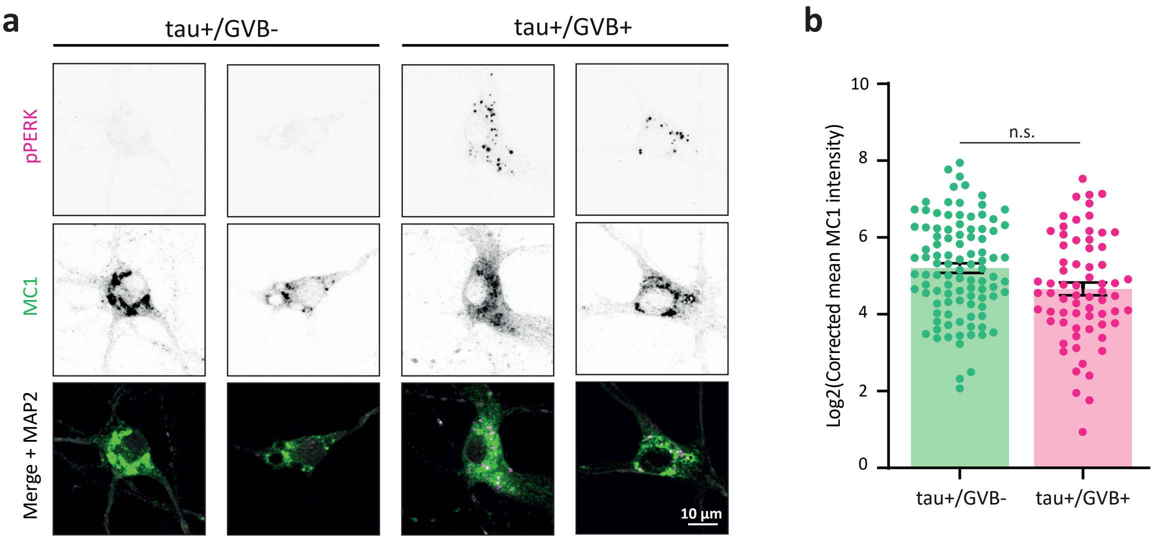

Supplementary Figure 3 Pathological tau levels are not different between GVB- and GVB+ neurons in tau/GVB model. a Representative confocal images of different GVB- and GVB+ neurons in the seed-independent tau pathology model. Immunofluorescence staining was performed for MAP2 (grey) for neuron visualisation, for the GVB marker pPERK (magenta) and MC1 (green). Separate channels are shown in greyscale. **b** Single-cell quantification of somatic tau load represented as the Log2-transformed corrected mean MC1 intensity. Data points represent single neuron values. Error bars represent standard error of the mean (SEM). N=4 independent experiments, n=100 and 68 for tau+/GVB- and tau+/GVB+ populations, respectively. n.s.=not significant, nested t-test.

Supplementary Figure 4

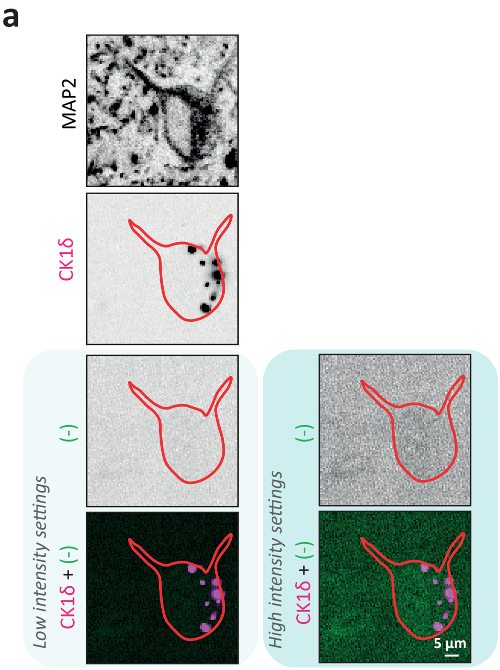

Supplementary Figure 4 Negative staining control for Figure 3. a Representative confocal images of a GVB+ neuron in the hippocampal tissue of a tauopathy patient. Immunofluorescence staining was performed for MAP2 (grey, delineated in red in the rest of panels) and CK1δ (magenta), while AT8 primary antibody was omitted (green). Separate channels are shown in greyscale.

Supplementary Figure 5

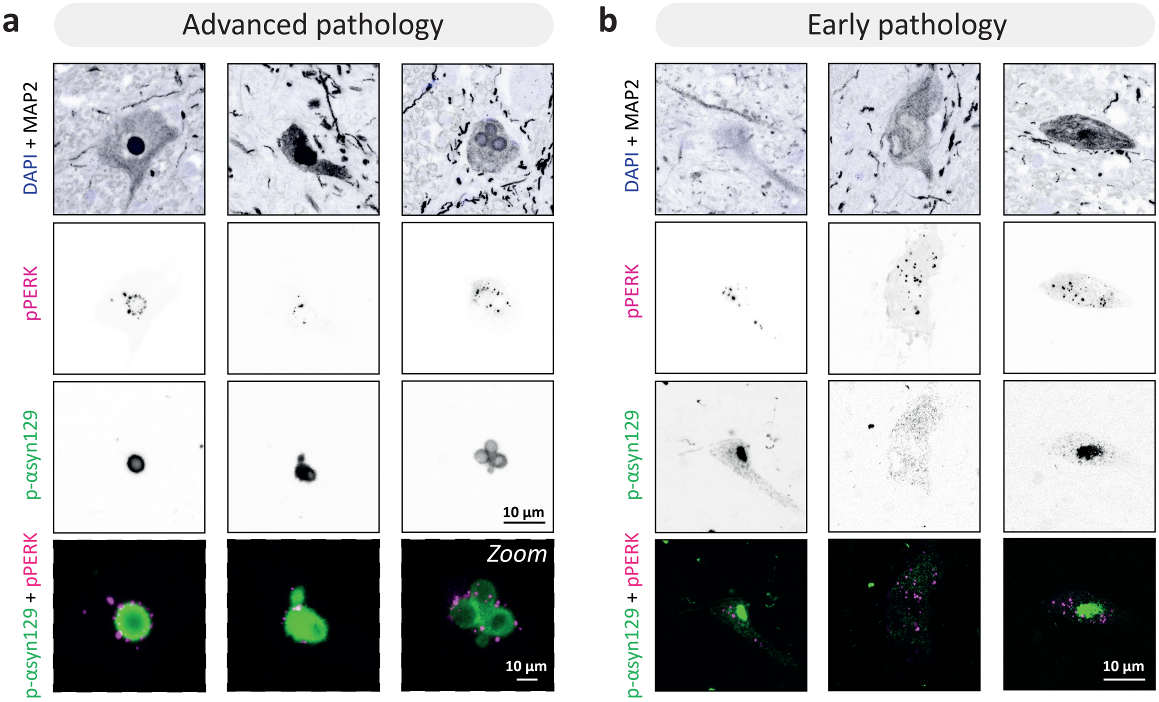

Supplementary Figure 5 Characterisation of GVBs in human PD *substantia nigra*. a,b Representative confocal images of GVB+ neurons in PD SN tissue derived from different patients. Immunofluorescence staining was performed for MAP2 (grey) for neuron visualisation, for the the GVB core marker pPERK (magenta) and p-αsyn129 (green). Nuclei are visualised by DAPI (blue), shown in an inverted merge with MAP2. Separate channels are shown in greyscale. **a** Examples of neurons with advanced α-syn pathology are shown. A zoomed merge is provided for better visualisation of the distribution of GVBs found in association with LBs. **b** Examples of neurons with early α-syn pathology are shown. Distribution of GVBs in these neurons resembles that of tau-induced GVBs in the AD brain.

Supplementary Figure 6

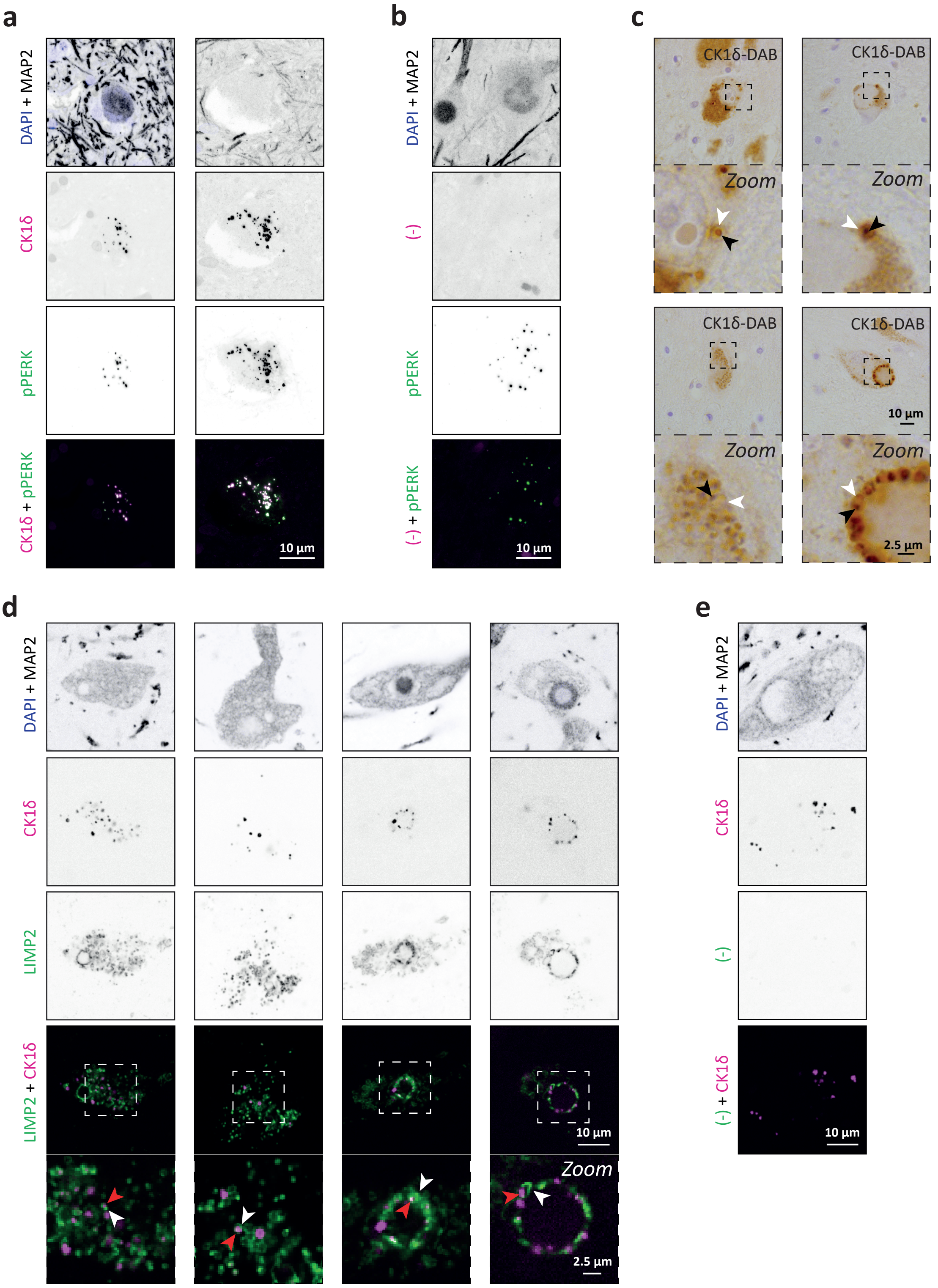

Supplementary Figure 6 Validation of GVB identity in human PD *substantia nigra*. a-e Representative confocal images of GVB+ neurons in PD SN tissue derived from different patients. Neurons are visualised by MAP2 immunofluorescence (grey) and nuclei are visualised by DAPI (blue), shown in an inverted merge. Separate channels are shown in greyscale. **a** Co-immunofluorescence for the canonical GVB marker CK1δ (magenta) and the additional GVB core marker pPERK (green) shows high colocalisation. **b** Staining control for (a) excluding CK1δ primary antibody. **c** Examples from different patients of immunohistochemical DAB staining of CK1δ and haematoxylin to show the typical GVB morphology. Please note the presence of endogenous neuromelanin in addition to the DAB signal. The white arrowhead in the zoom points to the GVB membrane and the black arrowhead points to the GVB core. **d** Co-immunofluorescence for CK1δ (magenta) and the lysosomal membrane marker LIMP2 (green). White arrowheads point to the LIMP2-positive membrane and red arrowheads point to the CK1δ-positive GVB core. **e** Staining control for (d) excluding LIMP2 primary antibody.

Supplementary Figure 7

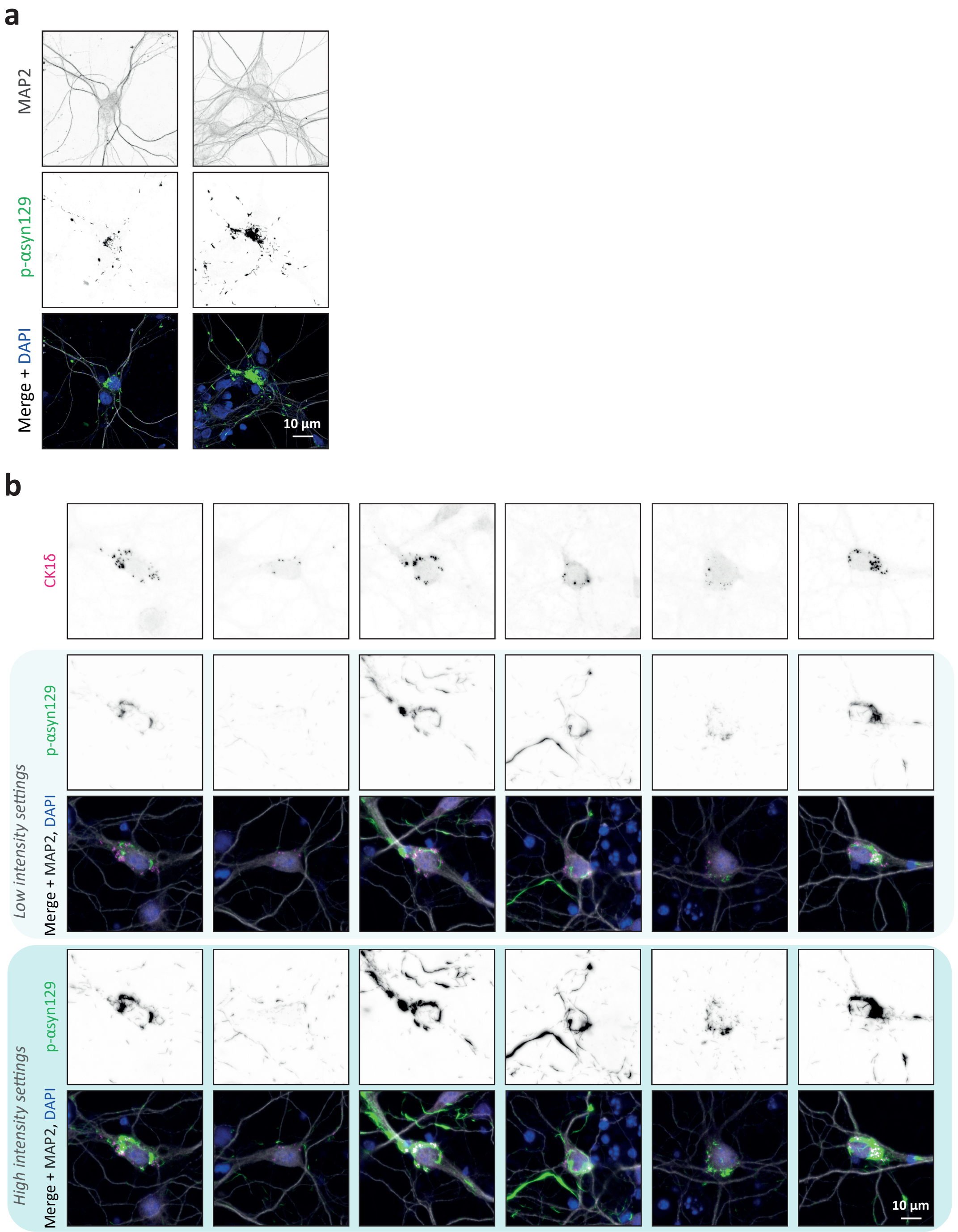

Supplementary Figure 7 Characterisation of the pathology induced in the α-synuclein seeded model. a Representative confocal images of MeOH-fixed neurons following the same experimental setup as in Fig. 5a. Immunofluorescence staining was performed for MAP2 (grey) and p-αsyn129 (green). Nuclei are visualised by DAPI (blue). Separate channels are shown in greyscale. **b** Representative confocal images of GVB+ neurons in the α-synuclein seeded model displaying different levels of pathology. Immunofluorescence staining was performed for MAP2 (grey), CK1δ (magenta) and p-αsyn129 (green). The p-αsyn129 signal is also shown at increased intensity to visualise low abundant pathological assemblies. Separate channels are shown in greyscale.

Supplementary Figure 8

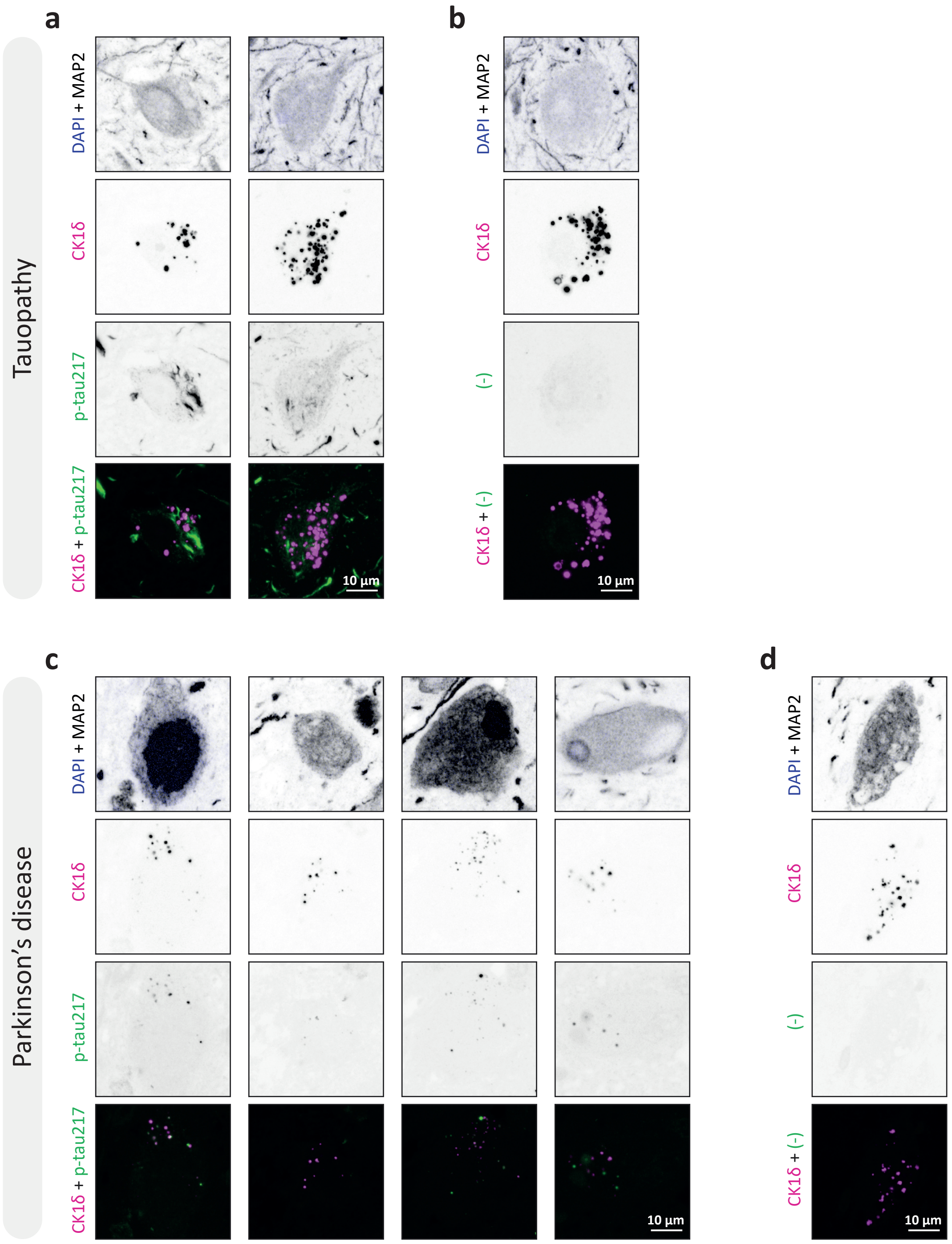

Supplementary Figure 8 P-tau217 immunofluorescence in human brain tissue. **a-d** Representative examples of GVB+ neurons derived from the different patients analysed. Immunofluorescence staining was performed for MAP2 (grey) and DAPI (blue), shown in an inverted merge, to visualize neurons and nuclei, respectively. Additionally, CK1δ (magenta) was used to detect GVBs and p-tau217 or the absence of it in control conditions is shown in green. Separate channels are shown in greyscale. **a, b** Examples of neurons found in the hippocampus of tauopathy patients. In this context, p-tau217 localises to the abundant cytosolic tau pathology (a) while the conditions in which no primary antibody for p-tau217 was added show no signal (b). **c, d** GVB+ neurons in SN of PD patients show overlapping signal between CK1δ puncta and p-tau217 which, in contrast to the tauopathy tissue, only appears in a punctate form (c). Control conditions in which p-tau217 primary antibody was omitted show no signal in green (d).

Supplementary table 1: Overview of independent experiments or patients and analysed cells per figure

Figure	Experiment detail	Number of independent experiments or patients	Number of cells analysed
Fig. 1b	pPERK + MC1	>4	>100
Fig. 1c	CK1δ (ms) + pPERK	3	110
Fig. 1d	CK1δ (ms) + LIMP2	3	64
Fig. 1e	pPERK + MC1	3	tau?/GVB+: 60<br>tau-/GVB-: 55
Fig. 2	CK1δ (rb) + MC1	3	0 days: 5566<br>1 day: 5037<br>4 days: 5655<br>8 days: 5515<br>11 days: 5450<br>13 days: 5477<br>14 days: 5775<br>15 days: 5540
Fig. 3	CK1δ + AT8	4	tau?/GVB+: 199<br>tau-/GVB-: 151
Fig. 4a & Sup. Fig. 5a,b	pPERK + p-αsyn129	4	43
Fig. 4b & Sup. Fig. 6a	CK1δ (ms) + pPERK	3	21
Fig. 4c & Sup. Fig. 6c	CK1δ-DAB	5	22
Fig. 4d & Sup. Fig. 6d	CK1δ (ms) + LIMP2	5	40
Fig. 5b & Sup. Fig. 7b	CK1δ (rb) + p-αsyn129	3	19
Fig. 5c	CK1δ (ms) + pPERK	3	8
Fig. 5d	CK1δ (ms) + LIMP2	4	15
Fig. 6a	pPERK + MC1	3	14
Fig. 6b,c	pPERK + AT8	3	Control: 64<br>α-syn PFFs/GVB+: 17<br>α-syn PFFs/GVB-: 123
Fig. 7a	CK1δ (rb) + p-tau217	3	92
Fig. 7b	CK1δ (rb) + p-tau217	3	47
Sup. Fig. 2a	MC1 MeOH	3	100
Sup. Fig. 2b	pPERK + AT100	1	122
Sup. Fig. 3	pPERK + MC1	4	tau+/GVB-: 100

			tau+/GVB+: 68
Sup. Fig. 7a	CK1δ (rb) + p-tau217	2	118
Sup. Fig. 7c	CK1δ (rb) + p-tau217	4	50
